# Deciphering the impact and mechanism of Trikatu, a spices-based formulation on alcoholic liver disease employing network pharmacology analysis and *in vivo* validation

**DOI:** 10.3389/fnut.2022.1063118

**Published:** 2022-11-16

**Authors:** Ruchi Sharma, Mangala Jadhav, Neha Choudhary, Arun Kumar, Abdur Rauf, Rohit Gundamaraju, Abdullah F. AlAsmari, Nemat Ali, Rajeev K. Singla, Rohit Sharma, Bairong Shen

**Affiliations:** ^1^Department of Rasa Shastra and Bhaishajya Kalpana, Faculty of Ayurveda, Institute of Medical Sciences, BHU, Varanasi, Uttar Pradesh, India; ^2^Department of Rasa Shastra and Bhaishajya Kalpana, R. A. Podar Ayurvedic Medical College, Mumbai, India; ^3^Centre for Computational Biology and Bioinformatics, Central University of Himachal Pradesh, Dharamsala, Himachal Pradesh, India; ^4^Institute of Nuclear Medicine and Allied Sciences (INMAS), Defence Research and Development Organisation (DRDO), New Delhi, India; ^5^Department of Chemistry, University of Swabi, Anbar, Pakistan; ^6^ER Stress and Mucosal Immunology Lab, School of Health Sciences, College of Health and Medicine, University of Tasmania, Launceston, TAS, Australia; ^7^Department of Pharmacology and Toxicology, College of Pharmacy, King Saud University, Riyadh, Saudi Arabia; ^8^Institutes for Systems Genetics, Frontiers Science Center for Disease-Related Molecular Network, West China Hospital, Sichuan University, Chengdu, China

**Keywords:** ethanol-induced hepatotoxicity, hepatoprotective, herbal formulation, network pharmacology, *Piper longum*, *Piper nigrum*, Trikatu Churna, *Zingiber officinale*

## Abstract

*Trikatu Churna* (*TC*) comprising *Zingiber officinale* rhizome, *Piper longum*, and *Piper nigrum* fruit, is effective in treating liver diseases and has high nutraceutical values. However, the efficacy of *TC* in treating alcoholic liver disease (ALD) and its mechanism remain largely unknown. This study evaluated the hepatoprotective effects of different doses of *TC* as well as to identify the bioactive components and determine their mechanism of action against ethanol-induced ALD. A compound-target network analysis model of *TC* was established to identify its potential bioactive compounds and pathways that might regulate its hepatoprotective effects. Further, *in-vivo* studies were performed to validate the potential of *TC* (200 mg/kg and 400 mg/kg b.w.) in the treatment and management of ALD. The study revealed that both the dosages of *TC* demonstrate significant (*p* > 0.0001) hepatoprotective effects by improving body weight, total bilirubin, serum glutamic oxaloacetic transaminase (SGOT), serum glutamic pyruvic transaminase (SGPT), serum alkaline phosphate (ALP), total cholesterol, total protein, globulin, albumin, and liver morphology. The High-performance thin-layer chromatography (HPTLC) fingerprinting of *TC* showed the presence of piperine. Network pharmacology identifies the role of *TC* in regulating various signaling processes including Advanced glycation end products-receptor for advanced glycation end products (AGE-RAGE), Hypoxia-inducible factors (HIF-1), Nuclear factor kappa-light-chain-enhancer of activated B cells (NF-Kappa B), and Phosphatidylinositol 3-kinase/protein kinase B (PI3K/Akt) signaling to exert its anti-inflammatory, antioxidant and anti-apoptotic role in managing ALD. Based on the bioinformatics analysis, some of the key targets of *TC* were found to be Prostaglandin-Endoperoxide Synthase 2 (PTGS2) or Cyclooxygenase-2 (COX-2), Sirtuin 1 (SRT1), and caspase-3. These effects may serve as a novel therapeutic option for the treatment of ALD. These preclinical validation studies for the ethnopharmacological potential of *TC* in ALD treatment further paved the way for researchers to perform next-level translational and clinical studies. Further, in-depth experimental studies for the validation of these bioinformatics-based results will give a clearer picture of mechanisms.

## Graphical Abstract

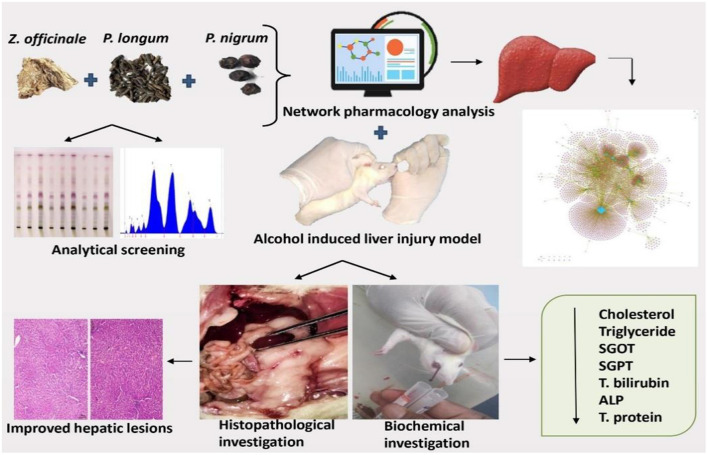

## Introduction

Globally, liver diseases and cirrhosis account for 23.59% of deaths, placing them 27th among all of the leading causes of death (1). In India alone, liver disease and cirrhosis make up to 2.74% of all causes of death (1). Globally, two main types of liver disease exist; alcoholic liver disease (ALD) and non-alcoholic fatty liver disease (NAFLD) (2). In India, alcoholism is the leading cause of chronic liver disease (3). According to WHO estimates, 140 million people worldwide suffer from alcohol dependence, and the disease is responsible for 3.8% of global mortality and 4.6% of disability-adjusted life years (DALYs) (4). ALD is a consequence of liver damage caused by acetaldehyde build-up and oxidative stress produced by alcohol consumption (5, 6). It includes manifestations of liver damage, such as fatty liver, alcoholism, or chronic hepatitis with cirrhosis or fibrosis (5, 6).

Further, no reliable hepatoprotective agent has been found to stimulate the liver's functions efficiently and protect against liver injuries with less toxicity (7). Plant-based hepatoprotective remedies to treat liver disorders have therefore received adequate attention on a global scale (8). A herbal formula like *Trikatu Churna* (*TC*), aka *Triushna* and *Vayosh* in Ayurveda, is derived from fine (#80) powder of three popular herbs, namely *Zingiber officinale* (*Z. officinale*) rhizome, *Piper longum* (*P. longum*) and *Piper nigrum* (*P. nigrum*) fruit (7). These three herbs are in use for thousands of years in the Indian traditional medical system to manage liver diseases (7).

These herbs have a similar bitter taste, but they are used differently. *TC* herbs are used in folk medicine for treating fever, asthma, diabetes, nasal diseases, obesity, anorexia, respiratory diseases, skin diseases, jaundice, and alcohol indigestion (9, 10). Several scientific reports claim that *TC* has anti-helminthic, anti-bacterial, hypo-lipidemic, anti-inflammatory, bioenhancing, anti-arthritic, immunomodulatory, adaptogenic, anti-anorectic, antioxidant, and antitumor properties (11–21). As part of describing the treatment of liver disease, Ayurvedic texts mention numerous formulations, 50% of which contain *TC* or its constituents (22). Of all the three ingredients, *P. longum* and *P. nigrum* contain an alkaloid piperine as the chief constituent (22), which has shown significant hepatoprotective effects against tert-butyl hydroperoxide and carbon tetrachloride (CCl_4_)-induced hepatotoxicity in the intoxicated mice (23). In another study, ethanol extract of *TC* exhibits hepatoprotective activity against CCl_4_-induced hepatotoxicity (24). Therefore, all these aforesaid reports suggest promising hepatoprotective properties of *TC*.

Despite the fact that *TC* is used in traditional medicine to treat several chronic liver diseases, no scientific evidence has yet been established that how it will alleviate ALD. As part of our study, we applied network pharmacology approaches to further understand the *TC* mechanism of action and active potential components. The emerging field of network pharmacology integrates concepts from system biology and computational biology to study, predict and illustrate the interactions between numerous components and targets in herbs at a systemic and molecular level (25). The potential of this approach to explore the multi-targeting and synergistic rationale of Ayurvedic herbs is well established (26, 27). The networks show the interactions between *TC* components and their molecular targets with liver diseases. This pioneering research might provide new insights into the pharmacodynamics of Ayurvedic drugs like *TC* and help to identify new leads and targets related to various diseases. It is an effective tool for analyzing *in silico* Ayurvedic medicine systems to determine the scientific rationale, active ingredients, and reaction mechanisms, but also requires further experimental exploration (28).

Therefore, for the first time, Ayurvedic claims of *TC* hepatoprotective effects on chronic liver injury caused by ethanol have been validated experimentally by using two doses of *TC* in Wistar rats and network pharmacology.

## Materials and methods

### Network pharmacological analysis

#### Phytochemical dataset and active compound screening

**Figure 1:**
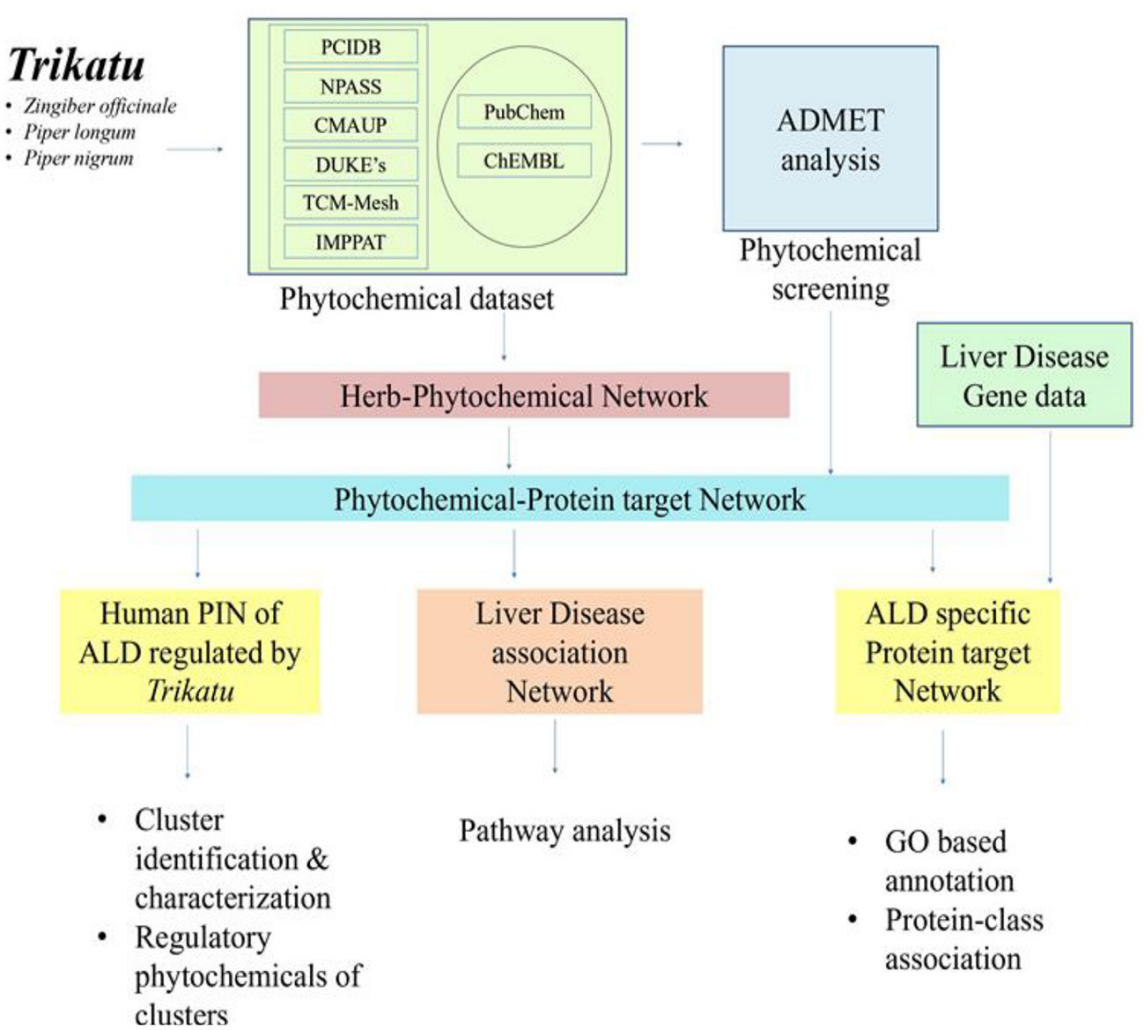
Methodology of network pharmacology.

The phytochemicals present in each constituent herb were compiled from six different database sources: CMAUP (29), Duke (30), IMPPAT (31), NPASS (32), PCIDB and TCM-mesh (33). The phytochemicals were checked for their chemical information from the PubChem database (http://www.ncbi.nlm.nih.gov/), and duplicate entries were removed to obtain a unique set of phytochemicals. Secondly, ADME parameters of the phytochemicals were acquired using FAFdrugs4 (http://fafdrugs4.mti.univ-paris-diderot.fr) (34). FAFdrugs4 is a free ADME-Tox filtering tool that is conductive in screening our compounds. The phytochemicals were screened on the basis of a “drug-like soft” filter and referred to as potentially-active compounds (PACs) of *TC* (Figure 1).

#### Candidate protein targets of TC

The potential human protein targets of phytochemicals were retrieved from the STITCH database (35). For the selection, only those compound protein targets within the score ≥700 were compiled and used for constructing the interaction network to study their relationship. To facilitate subsequent analysis, the target names were converted to UniProt IDs using the UniProt database (https://www.uniprot.org), a universal protein database.

#### Liver disease-associated genes

Liver disease-associated genes were collected from DisGeNET database (36). Herein, 38 liver disease terms were used and annotated using Medical Subject Headings (MeSH) or UMLS vocabularies (Supplementary Table S1). Only datasets corresponding to the curated gene-disease pairs were used in the present study. The target genes were labeled by using their Uniprot IDs.

#### Network construction and analysis

The network construction, visualization, and investigation were performed using an open-source software of Cytoscape (version 3.0) (37). For the network analysis, the Network analyzer utility of the Cytoscape was used.

Protein-protein interactions (PPIs) are central to the proper functioning of the most basic molecular mechanisms underlying cellular life and are often disturbed in disease states. To elucidate the regulatory effect of *TC* on PPIs associated with ALD, a protein interaction network (PIN) was derived from STRING, within the confidence, score range ≥900 (38). The Molecular Complex Detection (MCODE) algorithm was used to cluster the PIN and organize proteins into various modules to examine module-regulation of *TC* as per the mentioned approach (39). The MCODE algorithm detects densely connected regions among the protein interaction networks and offers a directed mode for fine-tuning of clusters among the PINs. It also provides a controlled mode for fine-tuning of the clusters to examine their interconnectivity in detail (40). Gene ontology (GO) based annotation as well as protein-class annotation of the protein targets among the networks were carried out using PANTHER (41). KEGG-pathway enrichment analysis was done using Database for Annotation, Visualization, and Integrated Discovery (DAVID) (42).

### Experimental analysis

**Figure 2:**
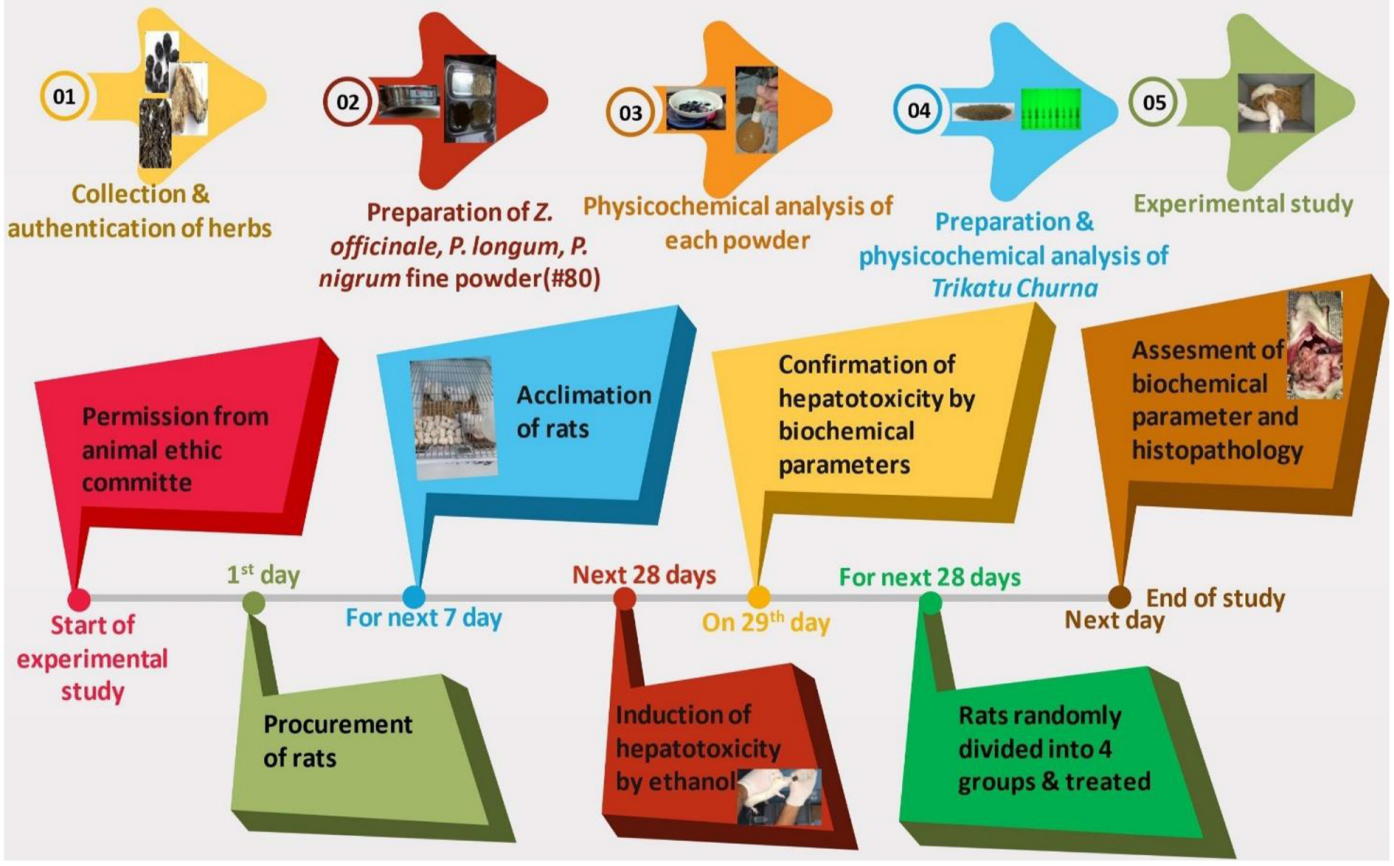
Protocol of the experimental study.

The plan of the experimental study is demonstrated in Figure 2.

#### Chemicals

We used silymarin, ethanol, saturated picric acid, and staining reagent hematoxylin and eosin (H&E) that were of the standard protocol and analytical grade (Sigma, USA). Biochemical parameters were analyzed by using an automated analyzer and diagnostic kits (ERBA, Czech Republic).

#### Collection and authentication of herbs

The crude herbs such as raw tuber/rhizome of *Z. officinale* and fruits of *P. longum* and *P. nigrum* were procured from the local market of Jammu, Jammu, and Kashmir, India and authenticated from the botanical institute of Savitribai Phule Pune University, Pune, India with specimen voucher number DNM/19. The samples were identified and authenticated using floras, taxonomic literature, macroscopic (external structure of the plant)/microscopic (palisade ratio, stomatal index, cell structure, epidermis, vascular bundle arrangement) characters. Their nomenclature is done as per IPNI (theplantlist.com).

#### Preparation and physicochemical analysis of testing formulation

The herbs were ground separately (#80) and blended in equal proportions to produce a homogeneous mixture. The prepared formulation was weighed and packed in an air-tight container under aseptic conditions (43). Physicochemical analysis of each herb and *TC* was done according to the Ayurvedic Pharmacopeia of India (API) guidelines. Physicochemical tests were performed to determine the foreign matter, moisture content, total ash value, acid insoluble ash, alcohol soluble extractive, and water-soluble extractive value (44). High-performance thin-layer chromatography (HPTLC) was performed to standardize and fingerprint *TC*. Based on the existing knowledge, chromatographic separation was carried out on HPTLC plates precoated with silica gel 60 F254 (E. Merck) of 0.2 mm thickness supported with aluminum sheet support. Plates were developed in a glass twin trough chamber (CAMAG automatic TLC sampler 4) that was pre-saturated with the mobile phase. The experimental condition was maintained at 20 ± 2°C. After derivatizing plates with anisaldehyde sulphuric acid reagent and photo documentation with CAMAG Reprostar 3 photo documentation platform at 550 nm, piperine could be detected. The solvent system consisting of toluene-ethyl acetate-glacial acetic acid (8:2:0.1, v/v/v) was used to resolve piperine from the matrix of *TC* (45–47). Densitometric determinations were performed in fluorescence mode by CAMAG TLC scanner.

#### Dosage fixation

For the present study, the doses of *TC* were calculated by extrapolating the maximum tolerated dose from existing scientific evidence on the toxicity of *TC*. *TC* at 2,000 mg/kg body weight once orally and 5, 50, and 300 mg/kg body weight once daily for 28 days were well tolerated by the Charles Foster rats (both male and female) (48). The safety of consuming the herb was also a great concern despite its potential, so all these evidence are crucial in justifying its safety. Also, a study on an ethanol extract of *TC* at an oral dose of 150 mg/kg exhibited a significant protective effect by lowering serum levels of glutamic oxaloacetic transaminase, glutamic pyruvic transaminase, alkaline phosphatase, and total bilirubin (24). By considering all these data from the previous studies, the two different dose groups of 200 and 400 mg/kg orally were considered for evaluation of the hepatoprotective activity.

#### Experimental design

An animal study was conducted at Agharkar research institute, Pune, India, according to the recommendations and approval of the Institutional Animal Ethical Committee with approval number ARI/IAEC/2019/11. A total of 24 male Wistar rats 6–8 weeks old (180–200 g) were procured and housed under standard environmental conditions (normal daily light cycle, 40–60% relative humidity and 25°C temperature). Animals were fed on a standard chow diet and provided with purified water *ad libitum*. In addition, animals were marked with a saturated picric acid solution in water for proper identification.

#### Ethanol-induced hepatotoxicity model in rats

Hepatotoxicity was induced by oral administration of ethanol once daily for 28 days. As tolerance developed, ethanol dosage was gradually increased in order to maintain blood alcohol levels between 150 and 300 mg/dl. The starting dose was 8 g/kg per day for 15 days and then increased gradually by 1 g/kg per day for the next 7 days and then final dose for the next 7 days and the rest of the study was 16 g/kg per day. Following 28 days, six rats in total were randomly selected and 2 ml of blood was collected under light anesthesia from the retro-orbital space of the eye for biochemical analysis: serum glutamic oxaloacetic transaminase (SGOT), serum glutamic pyruvic transaminase (SGPT), serum alkaline phosphate (ALP), total bilirubin, cholesterol, triglyceride, total protein, albumin, and globulin levels to confirm hepatotoxicity. After confirmation of toxicity, animals were randomly divided into four groups (each group containing 6 rats): control group (no treatment, only ethanol), Test drug 1 group (*TC* 200 mg/kg + ethanol), Test drug 2 group (*TC* 400 mg/kg + ethanol), Standard group (Silymarin 100 mg/kg + ethanol). Rats were weighed and given following treatment along with a standard diet for 28 days. A suspension of *TC* was made with distilled water for oral administration to the animals. Even after induction of hepatotoxicity, in the dosing schedule, ethanol is not stopped and is given to the control group and treatment groups along with the treatment to maintain the state of hepatotoxicity as it is known that liver has tendency to regenerate by itself also. Total duration of study is for 56 days (28 days for induction of hepatotoxicity and 28 days for treatment schedule).

#### Biochemical investigation and histopathology

On the 29th day of treatment, under light anesthesia, 2 ml of the blood was conveniently collected from the retro-orbital space of each rat and sent for biochemical investigations. The serum from the blood was separated by centrifuging it at 3,000 rpm, 4°C for 10 min and used for measurement of the various biochemical markers SGOT, SGPT, ALP, total bilirubin, cholesterol, triglycerides, total protein, albumin, and globulin. Furthermore, rats of each group were sacrificed by giving a 50 mg/kg dose of pentobarbital intraperitoneally, and then the dissection was done. The slices of the left liver lobe and left kidney (from six animals of each group) were removed and fixed in 10% formalin for 24 h. After 24 h, tissues were embedded in paraffin and 5–6 μm sections were routinely stained with hematoxylin and eosin (H&E) and assessed in a light microscope. All alterations from the normal structure were registered.

#### Statistical analysis

Results were presented as mean ± SEM, the difference between the groups was statistically determined by one-way analysis of variance (ANOVA) followed by the Tukey-Kramer Multiple Comparisons Test. The comparison of weight before treatment but after induction of disease (BT) and after treatment at end of the study (AT) was statistically determined by paired t-test with a level of significance at *P* < 0.05. The level of significance was noted and interpreted accordingly.

## Results

### Network pharmacology-based analysis

For further exploring the global effects of *TC* and its potential role in managing ALD, a network pharmacology study was performed.

#### Phytochemical dataset of TC

**Figure 3:**
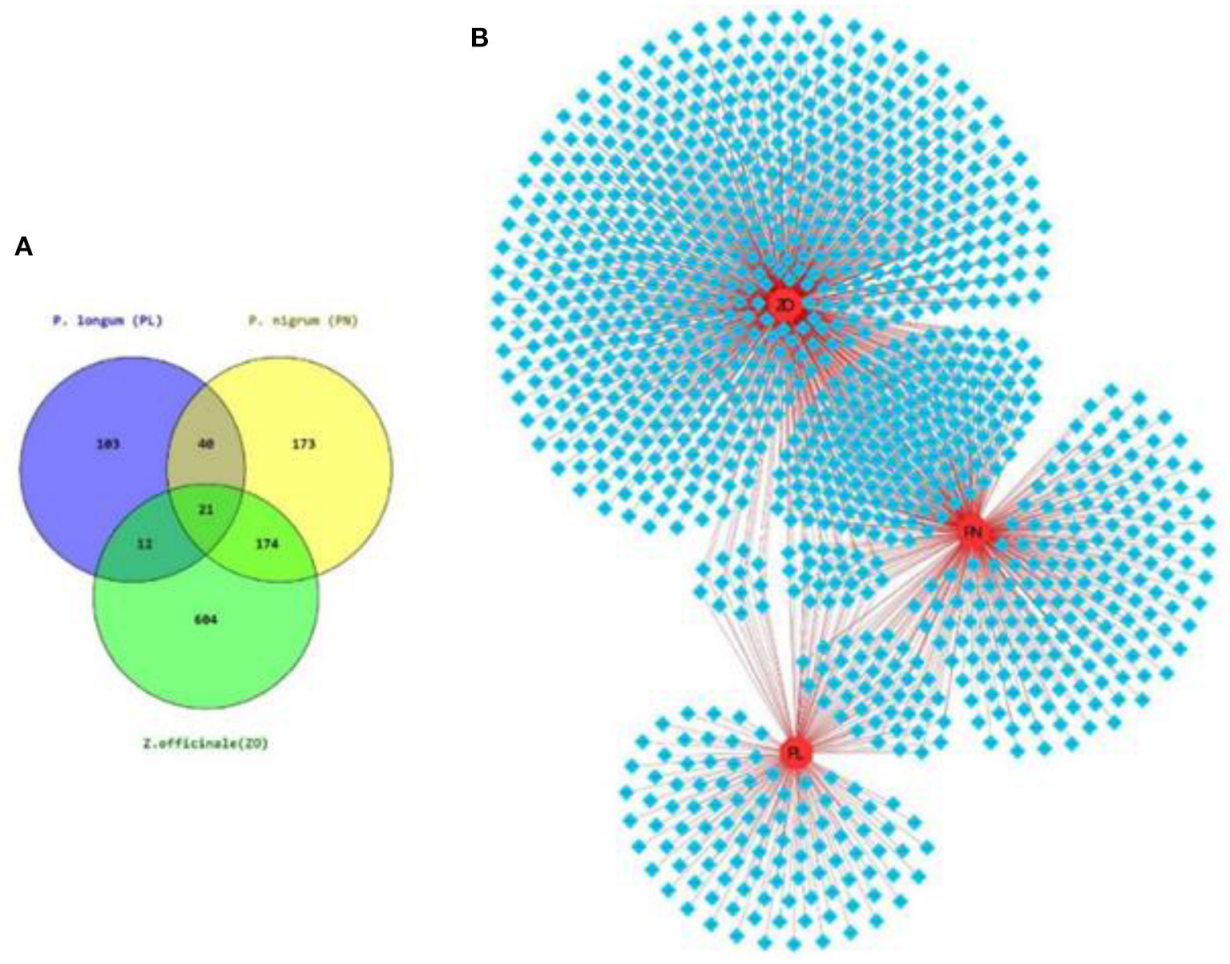
Phytochemical composition of *TC*. **(A)** Venn diagram showing the herb-wise distribution of 1,127 PCs associated with the tri-herbal formulation of *TC*. **(B)** Herb-Phytochemical Network of size 1,130 node 1,395 edges, where 1,127 PCs are shown in blue diamonds and herbs in red circular nodes.

A phytochemical search among the databases (mentioned in the Material and methods section) resulted in the compilation of 1,127 PCs in *TC* (Figure 3A).

The information of each herb of *TC* and their corresponding PCs was used to construct herb-phytochemical (H-PC) network (Figure 3B). The herb-phytochemical association along with the chemical information of each PC is given in Supplementary Table S1.

ADME analysis of the PCs using FAFdrugs4 leads to the selection of 418 PCs (referred to as PACs) when screened on the basis of a “drug-like soft” filter (Supplementary Table S1).

#### TC-protein target network

Human target proteins of *TC* were compiled from the STITCH database, which were further used to construct *TC*-protein target (T-PT) network. For the STITCH confidence score ≥700, 2,765 protein targets were obtained for 198 PCs, resulting in the T-PT network of size 2,963 nodes and 7,055 edges (Supplementary Figure S1; Supplementary Table S2).

**Figure 4:**
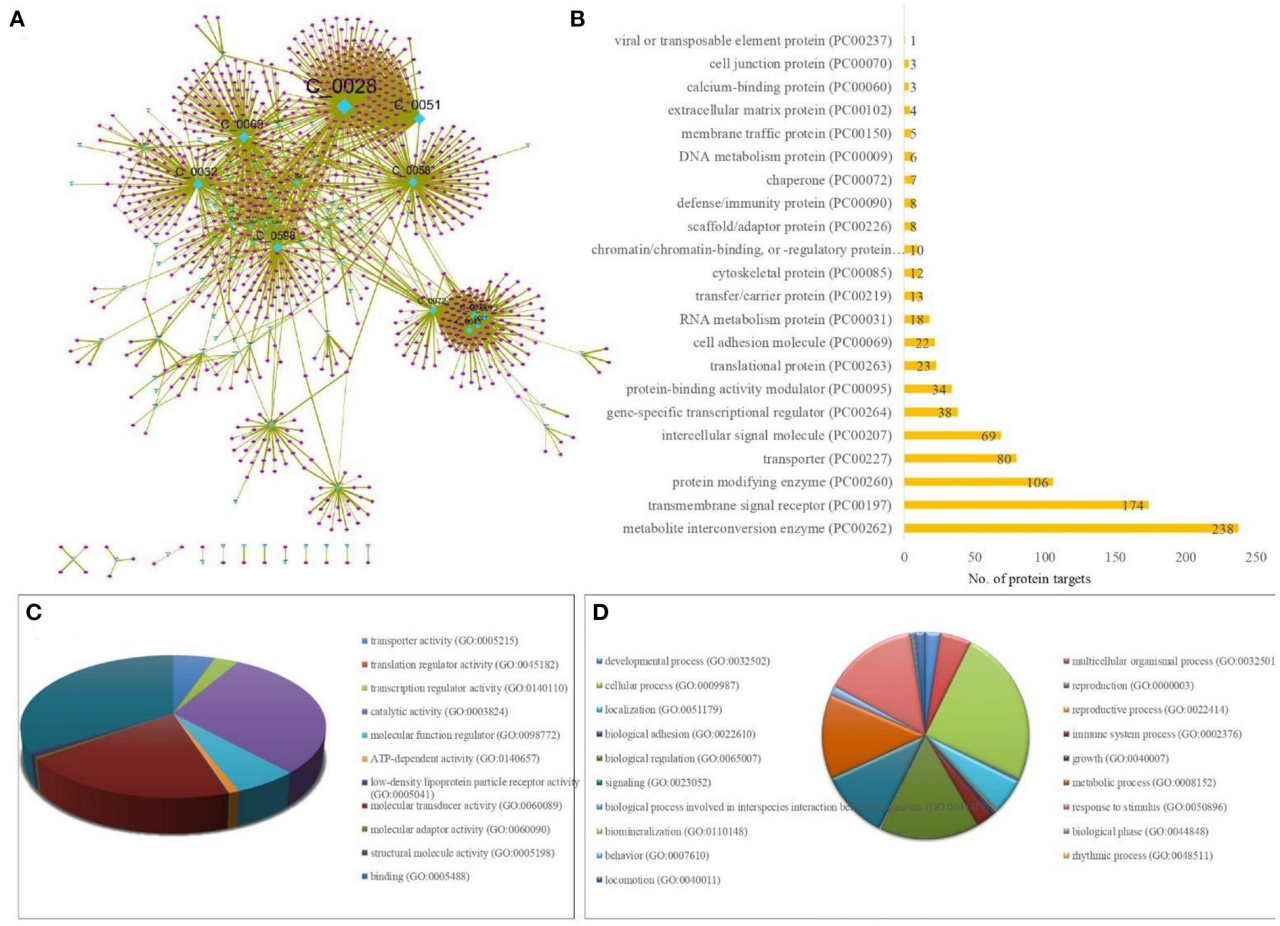
Protein targets of *TC* specific to PACs (potentially active compounds of *TC*). **(A)** Sub-network of size 1,100 nodes and 1,967 edges containing 95 PACs (cyan colored diamond nodes) and their 1,005 protein targets (pink colored circular nodes). The size of the nodes varies according to their degree value in the network, where the node with the highest degree is bigger in size corresponding to the other nodes. The protein targets were compiled from STITCH having interaction score ≥700. **(B)** Bar-graph showing the protein class association of the protein targets of PACs, highlighting the major contribution of the class of metabolite interconversion enzyme. X-axis represents the number of protein targets associated and the Y axis represents the protein-class. **(C)** GO-based molecular processes associated with protein targets of PACs with high enrichment among the class corresponding to “Binding (GO:0005488)” with involvement of 393 protein targets. **(D)** GO-based biological processes associated with protein targets of PACs with high enrichment among the class corresponding to “cellular process (GO:0009987)” with involvement of 641 protein targets.

A sub-network specific to PACs was derived from the T-PT network, to highlight the regulatory PACs and their specific human protein targets. This resulted in a sub-network of size of 1,100 nodes and 1,967 edges containing 95 PACs and their 1,005 protein targets (Figure 4A; Supplementary Table S3).

The protein targets of PACs majorly belonged to the class of metabolite interconversion enzyme, transmembrane signal receptor, and protein modifying enzymes (Figure 4B).

To decipher the biological classification of protein targets of PACs, gene ontology analysis was also carried out. As shown in Figures 4C,D, GO analysis indicated that the enriched molecular function corresponds to binding (GO:0005488) and catalytic activity (GO:0003824). For the biological process, the proteins were involved in the cellular process (GO:0009987), biological regulation (GO:0065007), response to stimulus (GO:0050896), metabolic process (GO:0008152), signaling (GO:0023052), and immune system processes (GO:0002376). GO-based annotation and the protein class association are detailed in Supplementary Table S3.

#### Liver disease-gene association network

Based on 38 liver disease terms and their associated curated gene-disease DisGeNET data, 516 unique proteins were harvested. The proteins and their associated liver disease term were used for obtaining the liver-specific disease gene (LD-G) network. In curated gene-disease pairs, only 24 of 38 liver disease terms could be obtained, which resulted in an LD-G network of size 539 nodes (515 proteins and 24 disease terms) and 1,308 edges (Supplementary Figure S2; Supplementary Table S4).

#### Relationship between TC targets and ALD genes

**Figure 5:**
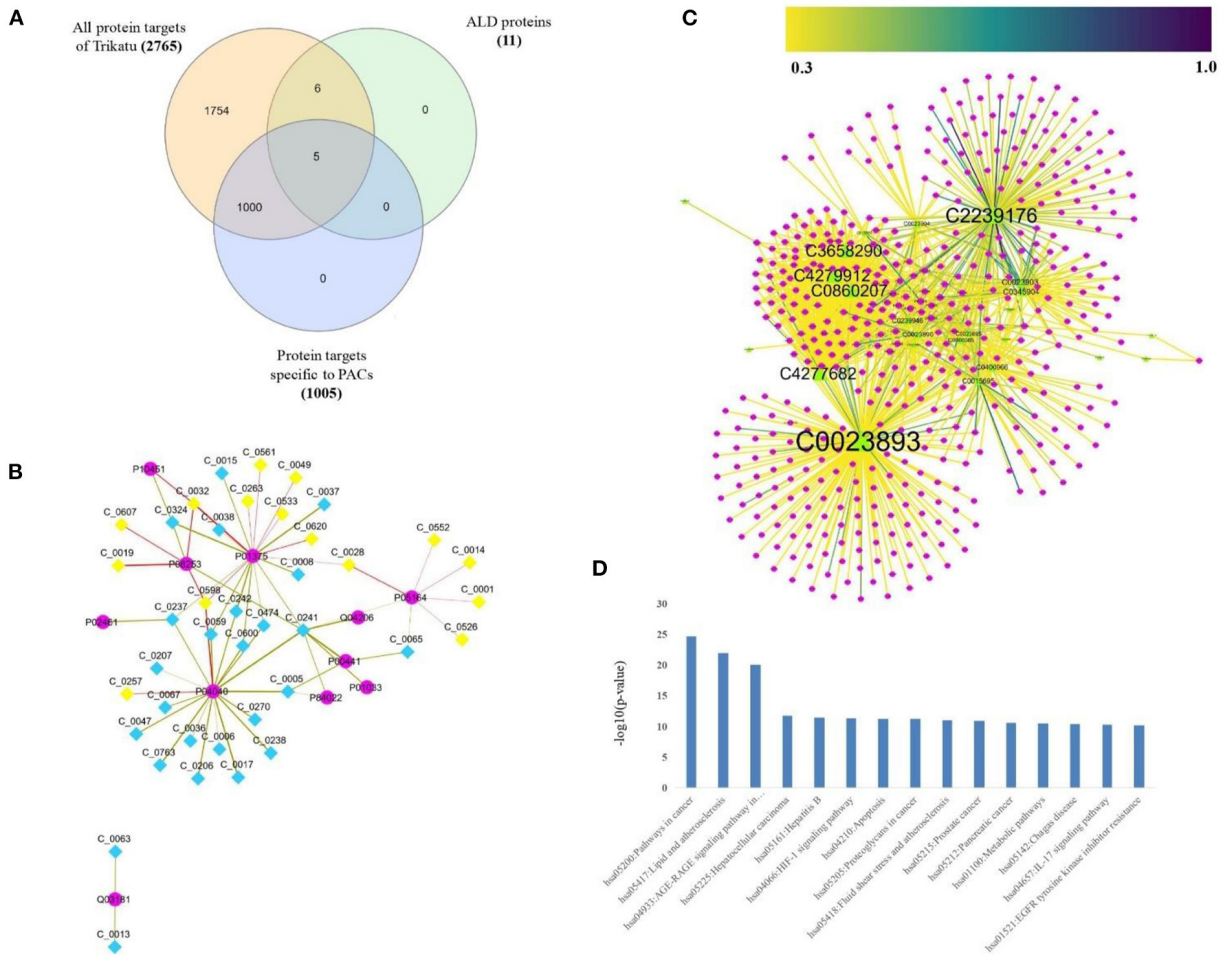
Regulatory phytochemicals of *TC* in managing ALD. **(A)** Venn-diagram showing the distribution of protein targets of *TC*. **(B)** Alcoholic Liver Disease-specific Protein Target (ALD-PT) network comprising of 51 nodes and 63 edges, consisting the information of 11 ALD proteins (diamond nodes) and their 40 regulatory (pink circular nodes) PCs of *TC*. Among the 40 PCs, yellow highlighted nodes represent PACs. **(C)** Protein-disease association network, highlighting the regulatory role of 40 PCs *via* their 453 protein targets (pink circular nodes) toward 24 liver-associated disease terms (green triangular nodes). The size of the nodes varies according to their degree value in the network. The edge color of the protein-disease pair varies as per the DisgeNET score. **(D)** Pathway association of the 453 protein targets of 40 PCs of ALD-PT network, where X-axis represent the Human-pathway and Y-axis represent the –log10 (*P* value). For the display only 15 highly enriched pathways based on *P*-value were selected, highlighting the major role of PCs in regulating cancer and signaling processes.

To explore ALD in detail, all the protein targets linked with ALD were derived from the LD-G network. The proteins were checked for their regulatory phytochemicals in the T-PT network and a sub-network specific to ALD was derived, which is referred to as ALD-PT network. The network consisted of 51 nodes and 63 edges, demonstrating the information of 11 ALD proteins (Figure 5A) and 40 PCs of *TC* (Figure 5B; Supplementary Table S5).

**Figure 6:**
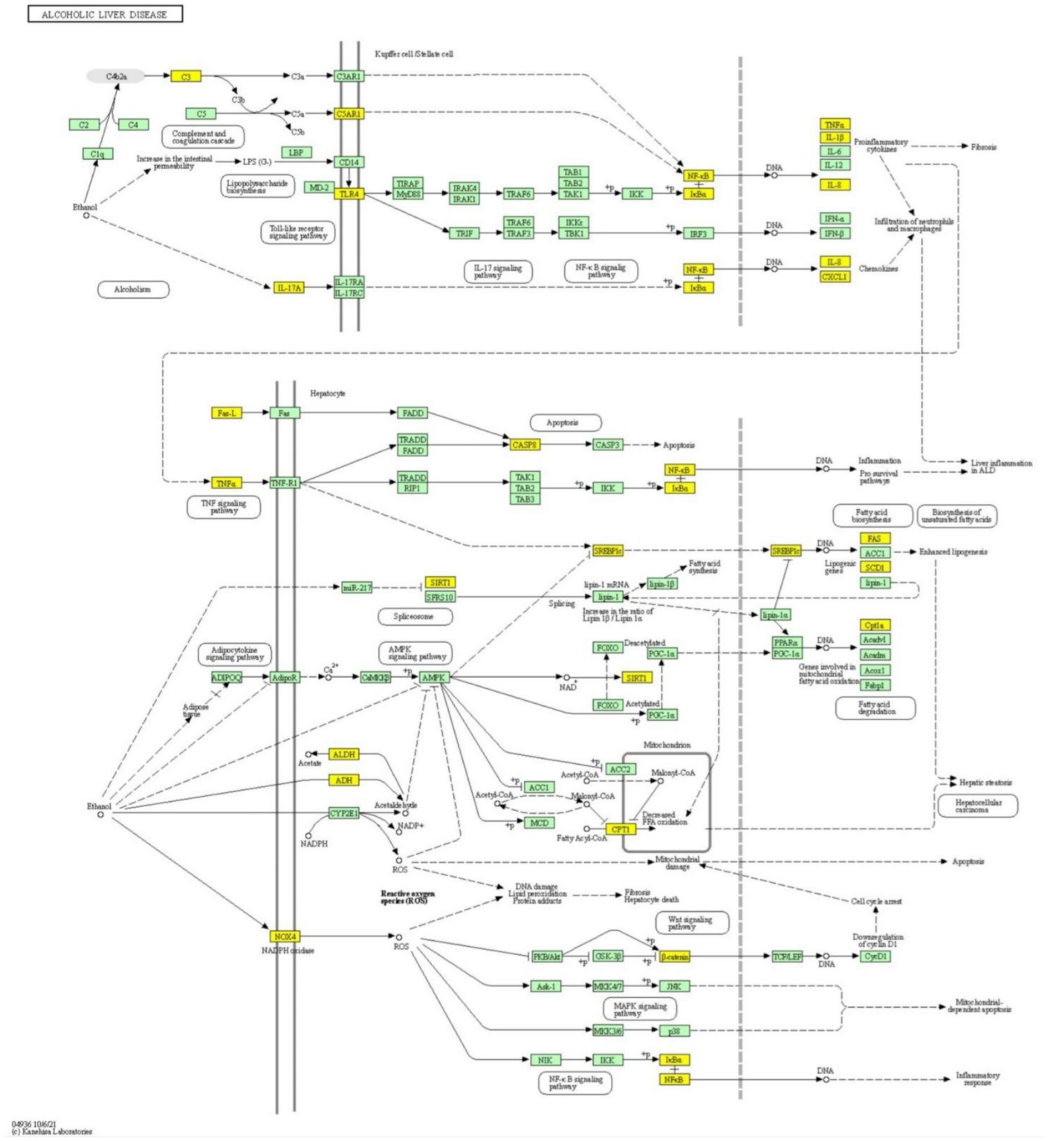
Pathway mapping of the *TC* protein targets in the ALD pathway. Mapping of the protein targets location of *TC* in the ALD pathway. The mapped proteins are highlighted in yellow boxes.

In addition to managing ALD, these 40 PCs also have the ability to regulate other liver diseases. This can be seen in Figure 5C, where 453 protein targets of these 40 PCs are linked with 24 liver disease terms. When checked for the pathway association of these proteins, the proteins were playing a major role in the pathways associated with cancer and signaling processes like advanced glycation end products **(**AGE-RAGE) signaling, Hypoxia-inducible factors (HIF-1) signaling pathway, interlukin-17 (IL-17) signaling, chemokine, and cytokine signaling, apoptosis signaling, Phosphatidylinositol 3-kinase**—**protein kinase B (PI3k-Akt) signaling pathway, etc (Figure 5D; Supplementary Table S6). Pathway analysis also highlighted the direct role of 26 proteins in the ALD disease pathway. The mapping of these proteins onto the ALD-disease pathway is given in Figure 6.

Among the disease association network, liver cirrhosis and liver carcinoma were identified as the major disease targets. It is interesting to note that 15 of these 40 PCs belong to PACs, further highlighting their role as lead molecules for future drug-discovery procedures against liver complications. These 15 PACs and their regulators can be checked in the ALD-PT network. The network topology attributes of the ALD-PT network were analyzed for their degree centrality measure, betweenness and closeness centrality. Among the network P01375 and P04040 were highlighted as nodes with high-degree centrality value of 19 each, reflecting high neighborhood connectivity of these nodes as degree centrality is a measure of number of connections a node of interest possesses. P01375 is a tumor necrosis factor encoded by TNFA gene, where TNF shows multifunctional properties for the liver heath including liver regeneration and inflammation, hepatocyte apoptosis and necroptosis, etc. (49). The other node among the network with highest degree value is P04040, a catalase protein encoded by CAT gene which is commonly expressed in liver. When checked for the betweenness centrality, a measure that quantifies the role of node to control the flow of information among the network, Q03181 and P01375 were showing high betweenness of 1 and 0.49 respectively (50, 51). Q03181 is a Peroxisome proliferator-activated receptor delta and play a significant role in liver health by acting as a major class of metabolic regulators (52).

Closeness centrality takes into account the average distance of all shortest path between a node to all other nodes among the network, where high closeness indicates high proximity to all other nodes. Among the ALD-PT network, Q03181 and C_0013 (1-butanol) were showing high closeness centrality values of 1.0 and 0.66, respectively (53). The degree, betweenness and closeness centrality values of each node among the ALD-PT network is provided in Supplementary Table S5. The information on multi-targeting PCs, including PACs in managing ALD among the ALD-PT network, obtained from network analysis has been shown in Table 1.

**Table 1:** The information of multi-targeting PCs, including PACs in managing ALD among the ALD-PT network.

**Serial no**.	**Phytochemical ID**	**Name of the compound**	**PAC** **(Yes or No)**	**No. of ALD targets**	**Associated plant**	**Part of the plant**
	C_0241	Zinc	N	8	*Z. officinale*	Rhizome
					*P. nigrum, P. longum*	Fruit
	C_0032	3-(1-Methylpyrrolidin-2-yl)pyridine	Y	3	*P. nigrum*	Fruit
	C_0237	Iron	N	3	*Z. officinale*	Rhizome
					*P. nigrum, P. longum*	Fruit
	C_0005	Acetaldehyde	N	3	*Z. officinale*	Rhizome
	C_0598	Quercetin	Y	3	*P. nigrum*	Fruit
					*Z. officinale*	Rhizome
	C_0324	Nicotine	N	3	*P. nigrum*	Fruit
	C_0600	Linoleic acid	N	2	*P. nigrum, P. longum*	Fruit
					*Z. officinale*	Rhizome
	C_0242	Silicon dioxide	N	2	*P. nigrum*	Fruit
	C_0065	Cysteine	N	2	*Z. officinale*	Rhizome
					*P. nigrum*	Fruit
	C_0474	Oleic acid	N	2	*P. nigrum, P. longum*	Fruit
					*Z. officinale*	Rhizome
	C_0028	Melatonin	Y	2	*P. nigrum*	Fruit
	C_0059	9,12-Octadecadienoic acid	N	2	*P. longum, P. nigrum*	Fruit

#### TC regulated human PIN (TRH-PIN) of ALD

**Figure 7:**
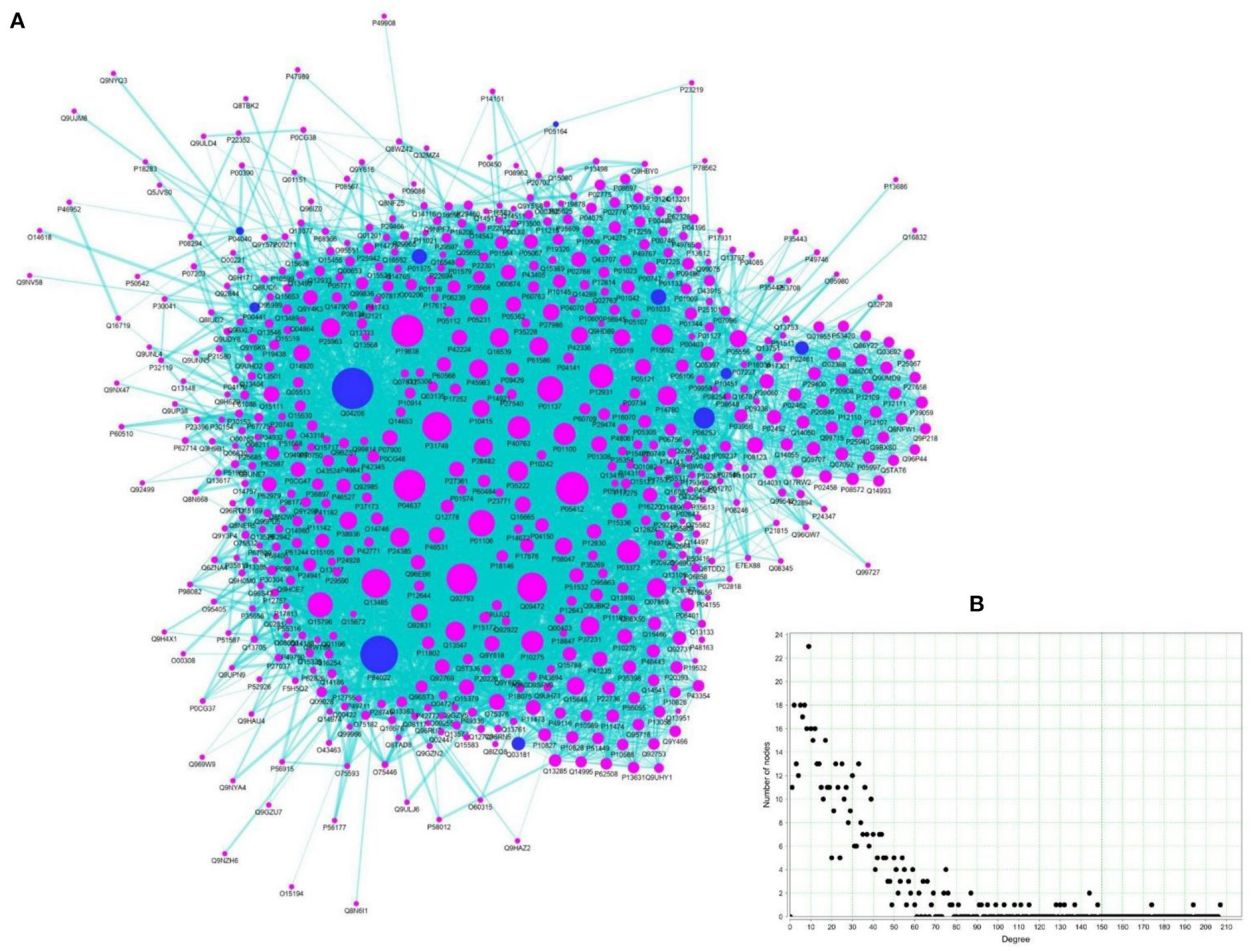
*TC* regulated Human Protein Interaction Network (TRH-PIN) of ALD. **(A)** Protein interaction network of 11 proteins of ALD (blue circular nodes), constructed from STRING with search limited to Homo sapiens and confidence score ≥900. The TRH-PIN consists of a network of size 580 nodes and 7,947 edges. **(B)** Node-degree distribution of TRH-PIN.

Toward elucidating the global effects of the *TC* in managing ALD, a PIN network of ALD-specific protein targets of *TC* was constructed and analyzed in detail. For this, high-confidence protein-protein interaction data of 11 proteins among the ALD-PT network was obtained from STRING *via* restricting the confidence score ≥900. This resulted in the compilation of 569 protein interactors of these 11 proteins leading to the network of size 580 nodes and 7,947 edges (Figure 7, TRH-PIN). The protein-protein interaction data of TRH-PIN along with the confidence score can be checked in Supplementary Table S7.

The information of topologically important nodes was also inferred for the TRH-PIN. Network analysis highlighted the role of P0CG48 and Q04206 as hub nodes among the PIN, owing to their high connectivity (or high degree value) with ability to regulate 207 and 194 other proteins, respectively. P0CG48 is a human Polyubiquitin-C protein encoded by UBC gene which is highly expressed in the liver and dysregulation of this gene is linked to defective proliferation of hepatocytes (54). The other hub-protein, Q04206 is a Transcription factor p65 encoded by RELA (NFKB3) gene responsible for master regulation of inflammatory and cell death processes with central link to the hepatocellular injury and liver fibrosis (51).

As already discussed, betweenness centrality measures the information-flow among the network. The nodes with high betweenness represent critical points of the network and are referred to as bottlenecks (55). Among the TRH-PIN, P0CG48 appeared as bottleneck with high betweenness centrality value of 0.10. Thus suggests the high relevance of this node in terms of its connectivity and control over the communication among the TRH-PIN. The degree and betweenness centrality values of each node among the TRH-PIN can be checked in Supplementary Table S7.

**Figure 8:**
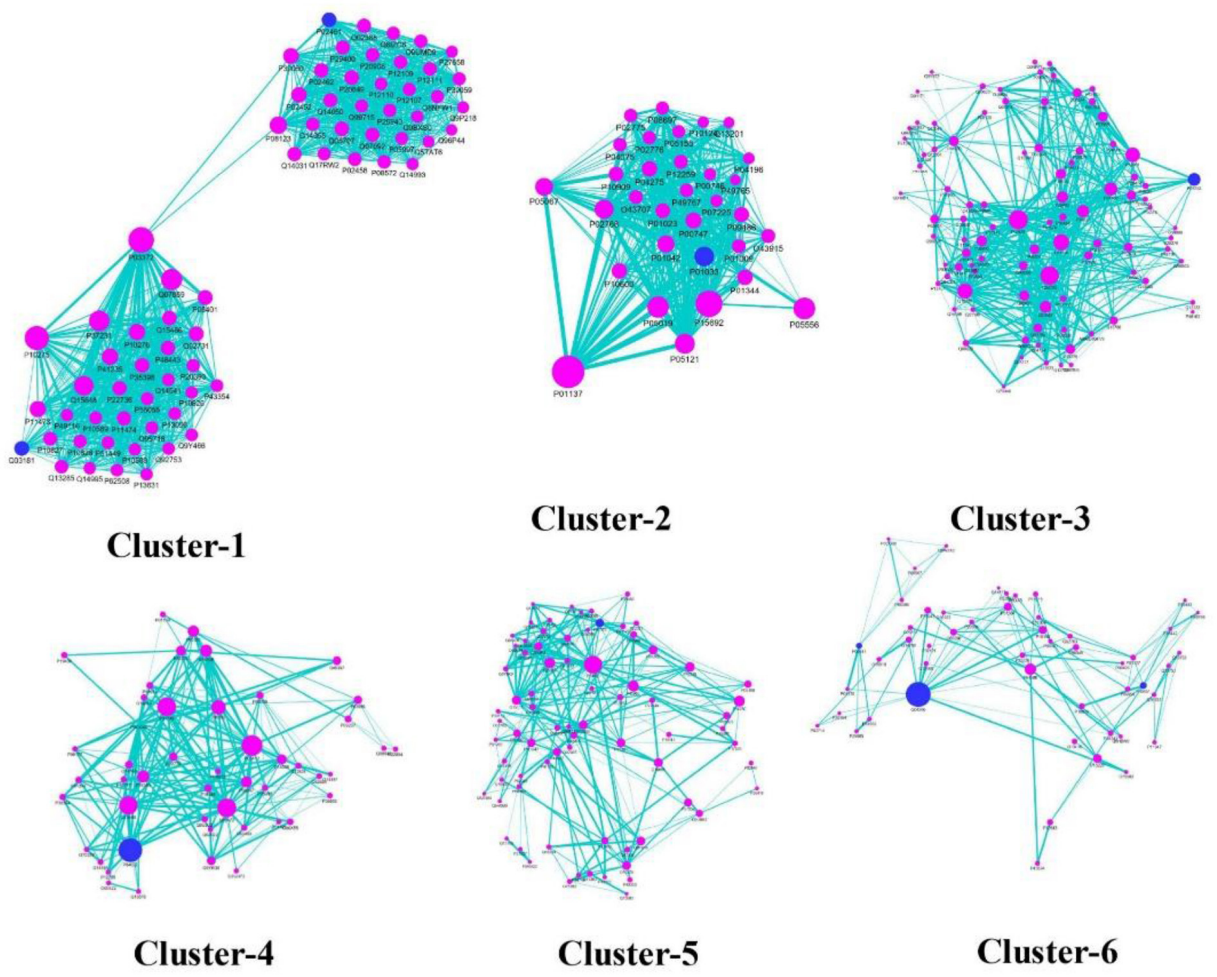
Cluster analysis of TRH-PIN. Six highly organized and connected topological clusters among the TRH-PIN. The location of ALD protein being targeted by phytochemicals of *TC* is shown in blue-colored nodes among the clusters, highlighting the direct regulatory role of *TC* in managing clusters.

To understand the modular architecture of the TRH-PIN of ALD, the network was subjected to module identification using MCODE. MCODE returned six highly organized and connected topological clusters (Figure 8; Table 2). To access the biological role of clusters, GO-based annotation of each cluster was carried out. The GO-based analysis highlighted the major association of proteins in molecular processes like binding (GO:0005488) and transcription regulator activity (GO:0140110). While the major biological processes were cellular processes (GO:0009987) and cellular anatomical entities (GO:0110165). The cluster proteins were showing their involvement among the pathways like PI3K-Akt signaling (hsa04151), Nuclear factor kappa-light-chain-enhancer of activated B cells (NF-kappa B) signaling (hsa04064), and pathways in cancer (hsa05200) especially hepatocellular carcinoma (hsa05225). A detailed description of the protein composition of each cluster and their associated GO annotation is given in Supplementary Table S8.

**Table 2:** MCODE cluster analysis of TRH-PIN of ALD with the information of regulatory phytochemicals of each cluster.

	**MCODE score**	**Nodes**	**Edges**	**Protein targets of *TC***	**Regulatory phytochemicals**	**Regulatory PACs**
Cluster-1	33.412	69	1136	20	31	11
Cluster-2	25.935	32	402	16	38	11
Cluster-3	11.677	94	543	41	75	36
Cluster-4	10.298	48	242	25	36	15
Cluster-5	7.6	76	285	37	60	23
Cluster-6	4.16	51	104	26	40	14

The regulatory phytochemicals of each cluster were also checked *via* searching for cluster proteins among the T-PT network. As a result, the regulatory phytochemicals in each cluster can be checked in Table 2. The multi-module regulatory potential of the PACs was also identified by searching the participation of protein targets of PACs among the clusters. This led to the identification of PACs like C_0032, C_0598 and C_0028 toward directly regulating the proteins of all the identified six-clusters, thus highlighting their high multi-module regulatory potential.

#### Physicochemical analysis

**Figure 9:**
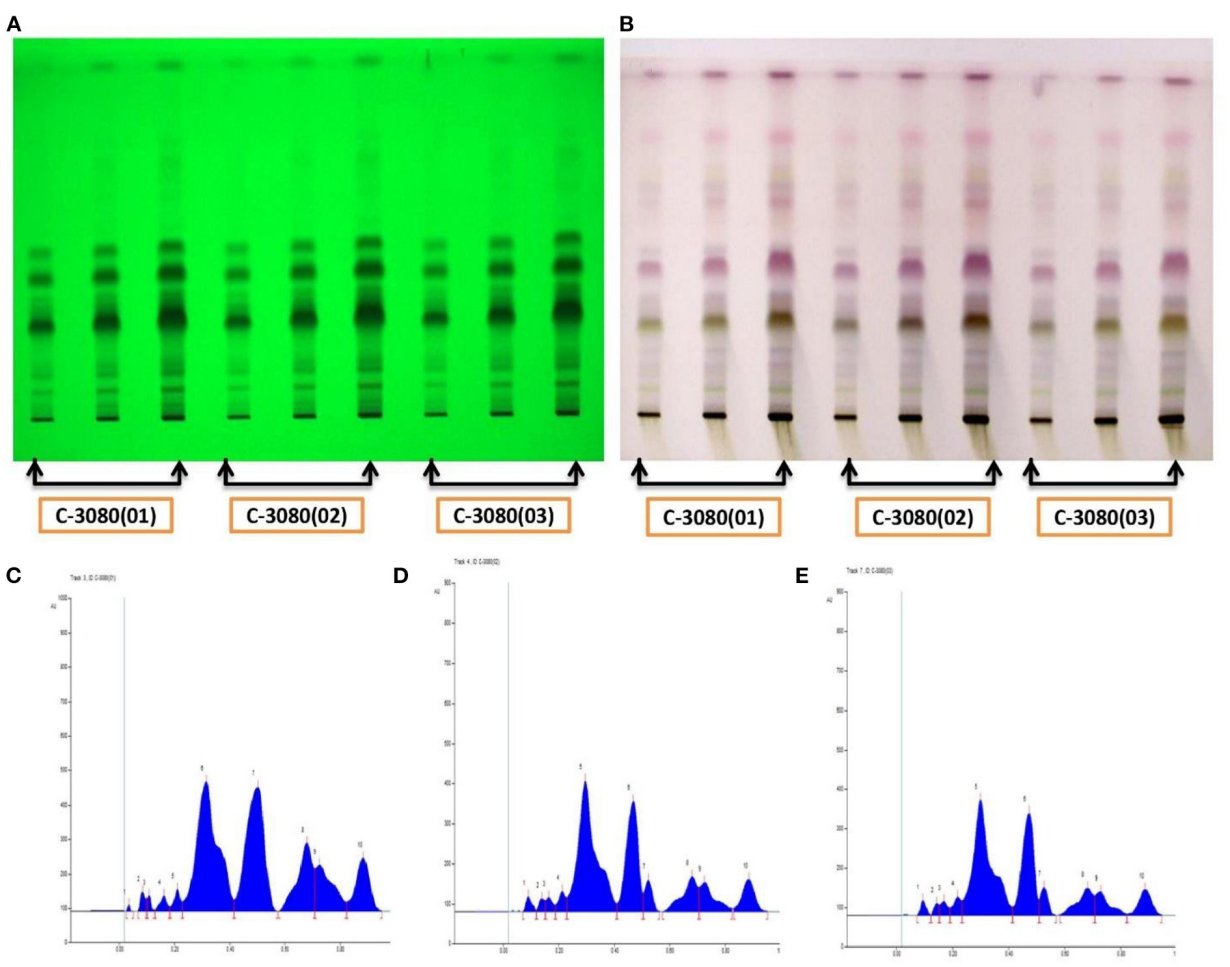
Fingerprinting and monographs of three batches of *TC*: Batch A [C-3080(01)], Batch B [C-3080(02)], Batch C [C-3080(03)], and by derivatization Reagent. **(A)** Fingerprinting of three batches of *TC* by using mobile phase Toluene: Ethyl acetate: Acetic acid (8:2:0.1) @ 254 nm, **(B)** Fingerprinting of three batches of *TC* by using derivatization reagent anisaldehyde sulphuric acid reagent @ 500 nm, **(C)** Peaks of batch A, **(D)** Peaks of batch B, **(E)** Peaks of batches C.

During powder preparation, the average mass yield% of *Z. officinale, P. longum*, and *P. nigrum* was 64.50, 63.20, and 88.57%, respectively. Organoleptic and physicochemical analyses of *Z. officinale, P. longum, P. nigrum* powder, and *TC* are recorded in Tables 3, 4, respectively. Results of physicochemical analysis of *TC* come under permissible values of pharmacopeial standards. Fingerprinting and monographs of HPTLC data of *TC* in triplicates were stipulated in Figure 9 and Table 5. Results indicate the presence of complex compounds. *Piperine* was found in *TC*.

**Table 3:** Organoleptic analysis of prepared herbal powder.

**Organoleptic tests**	***Z. officinale* powder**	***P. longum* powder**	***P. nigrum* powder**	***TC***
Sound	Not applicable	Not applicable	Not applicable	Not applicable
Touch	Rough	Rough	Rough	Rough
Color	Light yellow	Greenish black	Blackish brown	Light yellowish-brown
Taste	Pungent	Strongly pungent	Pungent	Astringent
Smell	Aromatic	Aromatic	Characteristic faint odor	Characteristic

**Table 4:** Physicochemical analysis of prepared herbal powder.

**Name of test**	***Z. officinale* powder**	**API standards**	***P. longum powder***	**API standards**	***P. nigrum powder***	**API standards**	***TC***
Moisture content	4%	NMT 5%	3.8%	NMT 5%	3.8%	NMT 5%	4%
Foreign matter	NIL	NMT 2%	NIL	NMT 2%	NIL	NMT 2%	**–**
Total ash	4.46%	NMT 6%	6.32%	NMT 7%	4.10%	NMT 5%	2.47%
Acid insoluble ash	0.83%	NMT 1.5%	0.21%	NMT 0.5%	0.30%	NMT 0.5%	0.61%
Alcohol soluble extractive	6.21%	NLT 3%	8.63%	NLT 5%	10.25%	NLT 6%	15.70%
Water soluble extractive	14.17%	NLT 10%	10.48%	NLT 7%	9.36%	NLT 6%	35.29%

NMT, not more than; NLT, not less than; API, Ayurvedic Pharmacopeia of India.

**Table 5:** HPTLC data—Batch A, B, and C of *TC*.

**Batch**	**Peak**	**Start position (Rf)**	**Start height (AU)**	**MAX position (Rf)**	**Max height (AU)**	**Max%**	**End position (Rf)**	**End height (AU)**	**Area (AU)**	**Area%**
A	1	0.03	0.8	0.04	19.5	1.34	0.06	0	144	0.22
	2	0.08	0.7	0.1	56	3.84	0.12	35.5	781.7	1.17
	3	0.12	36.5	0.13	45.8	3.15	0.15	3.6	527	0.79
	4	0.15	3.9	0.19	44.6	3.07	0.22	13.4	924.7	1.38
	5	0.22	13.8	0.25	63.9	4.39	0.27	27.5	1,142.6	1.71
	6	0.27	27.6	0.37	376.7	25.87	0.49	33	22,934.2	34.31
	7	0.49	33.3	0.59	360.8	24.78	0.67	0.1	18,912.9	28.29
	8	0.68	0.1	0.8	198.5	13.63	0.83	23.8	8,580	12.84
	9	0.83	123.9	0.85	134.8	9.26	0.97	26.4	6,659	9.96
	10	0.97	26.6	1.04	155.4	10.67	1.12	0.9	6,239.2	9.33
B	1	0.08	0.4	0.11	37.8	3.51	0.14	4.5	651.2	1.6
	2	0.14	5.5	0.16	32.1	2.99	0.18	27.6	541	1.33
	3	0.18	27.8	0.19	35	3.26	0.22	17.3	654.8	1.61
	4	0.22	17.6	0.25	50	4.65	0.27	35.7	1,001.7	2.47
	5	0.27	35.9	0.35	326.7	30.39	0.48	20.5	16,837.8	41.47
	6	0.48	20.7	0.55	274.8	25.57	0.59	45.7	9,577.1	23.59
	7	0.59	46.5	0.61	77.8	7.24	0.66	1	1,744.2	4.3
	8	0.67	0.1	0.8	87.5	8.14	0.83	60.4	3,677.9	9.06
	9	0.83	60.8	0.85	72.4	6.74	0.97	8.3	2,996.8	7.38
	10	0.97	8.2	1.04	80.7	7.51	1.12	0.8	2,915.4	7.18
C	1	0.09	0.1	0.11	37.1	3.84	0.14	1.8	590	1.65
	2	0.14	2.6	0.17	3.12	3.12	0.18	25.7	429.7	1.2
	3	0.15	25.8	0.2	3.64	3.64	0.22	18.7	720.3	2.01
	4	0.23	18.8	0.26	4.87	4.87	0.27	36.9	1,012.1	2.83
	5	0.28	37.1	0.35	30.2	30.2	0.49	21.3	15,008.7	41.89
	6	0.49	21.4	0.56	26.86	26.86	0.6	40.6	8,833.6	24.66
	7	0.6	41.6	0.62	7.32	7.32	0.67	0.1	1,557.4	4.35
	8	0.68	1.2	0.8	7.16	7.16	0.83	49.9	2,989.5	8.34
	9	0.83	49.9	0.86	6.21	6.21	0.96	3.4	2,281.7	6.37
	10	0.97	3.3	1.04	6.78	6.78	1.11	1	2,402.2	6.71

### Experimental analysis

#### Hepatotoxicity induced model

Induction of hepatotoxicity was confirmed, as all the biochemical parameters were raised from their normal range (Table 6).

**Table 6:** Results of biochemical parameters of six rats after induction of hepatotoxicity.

**Animal no**.	**SGOT**	**SGPT**	**Total bilirubin**	**ALP**	**Cholesterol**	**Triglyceride**	**T. protein**	**Albumin**	**Globulin**
1	85.6	65	0.55	198	164	123	6.1	3.1	4
2	120	72	0.6	181	213	153	6.6	2.87	3.6
3	90.2	49	0.76	156	157	122	5.9	2.9	4.1
4	110	65	0.82	210	232	120	5.4	3.2	3.9
5	126	77	0.76	169	311	116	6.9	3.6	3.2
6	98.12	51	0.52	198	298	145	8	2.8	3.2
Mean of the observation	104.9	63.1	0.67	185.3	229.1	129.8	6.48	3.08	3.6
Normal value of parameters	**45–80**	**17–35**	**0.3–0.7**	**120–180**	**120–160**	**60–120**	**4–8**	**2–5**	**2–4**

#### Body weight

Changes that took place in the weights of rats BT and AT from each group were statistically determined by Paired t-test, which are presented in Table 7. Mean and SEM represents the mean and standard error of the mean difference in animals of a group. It was found that treatment with *TC* and silymarin resulted in a significant weight increase in rats. However, on applying ANOVA (Table 8), no significant difference in the body weight of rats was found.

**Table 7:** Comparative data of before and after treatment for control, TD1, TD2, and standard group by applying paired t-test-body weight.

**Group**	**Mean differences** **±S. E. M of** **differences**	***P* value**	**Result**
Control	13.83 ± 17.75	>0.05	NS
TD1	47.33 ± 8.54	< 0.01	**
TD2	47.5 ± 13.69	< 0.05	*
Standard	62.5 ± 6.5	< 0.001	***

TD1, test drug 1; TD2, test drug 2; NS, not significant; ^*^ significant; ^**^ very significant; ^***^ highly significant.

**Table 8:** Comparative biochemical data of all groups (ANOVA test).

**Groups**	**Control**	**TD1**	**TD2**	**Standard**	**Results of ANOVA**
Body weight	277.67 ± 12.32	311.83 ± 7.56	304.50 ± 10.31	312.50 ± 11.901	NS
SGOT	119.67 ± 7.96	78.92 ± 3.43	53.62 ± 4.0	63.95 ± 13.57	***
SGPT	62.17 ± 8.03	44.83 ± 5.71	29.22 ± 3.99	31.12 ± 2.70	**
Total bilirubin	0.76 ± 0.04	0.58 ± 0.08	0.38 ± 0.05	0.33 ± 0.03	***
Sr. ALP	225.33 ± 12.06	171 ± 9.76	163.83 ± 8.58	144.83 ± 7.86	***
Cholesterol	263.17 ± 13.22	196 ± 10.24	153.33 ± 11.74	161.67 ± 9.47	***
Triglyceride	165.83 ± 6.90	102 ± 9.92	94 ± 11.39	111.83 ± 6.02	***
T. Protein	6.90 ± 0.17	5 ± 0.46	4.87 ± 0.59	4.68 ± 0.43	**
Albumin	4.32 ± 0.24	3.23 ± 0.28	3.03 ± 0.31	2.81 ± 0.48	*
Globulin	3.32 ± 0.27	2.45 ± 0.17	2.24 ± 0.21	2.53 ± 0.13	**

TD1, Test drug 1; TD2, Test drug 2; NS, not significant; Sr. ALP, serum alkaline phosphatase, T. Protein, total protein.

^*^ Significant; ^**^ very significant; ^***^ highly significant.

#### Biochemical parameters

The biochemical parameters of the different groups are expressed as mean ± SEM in Table 8 along with the statistically determined significance level of one-way analysis of variance (ANOVA). Further, the Tukey Krammer Multiple Comparisons test to compare treatment efficacy between the groups' results is performed and recorded in Table 9. *TC* at both doses shows highly significant potential in reducing SGOT, SGPT, ALP, total bilirubin, cholesterol, triglycerides, total protein, albumin, and globulin compared to the control group. But only, the higher dose of 400 mg/kg has shown a significant difference in reducing SGOT, SGPT, total bilirubin, cholesterol, total protein, albumin, and globulin parameters compared to the standard drug, which was less shown by *TC* (200 mg/kg) dose.

**Table 9:** Comparative results after applying Tukey-Krammer test between groups.

**Biochemical parameter**	**Control vs. TD1**	**Control vs. TD2**	**Control vs. standard**	**TD1 vs. TD2**	**TD1 vs. standard**	**TD2 vs. standard**
SGOT	< 0.00001	< 0.00001	< 0.00001	< 0.001	< 0.05	>0.05
	***	***	***	***	*	NS
SGPT	< 0.001	< 0.00001	< 0.01	< 0.001	< 0.01	>0.05
	***	***	***	***	**	NS
Total bilirubin	< 0.0001	< 0.00001	< 0.00001	< 0.0001	< 0.00001	>0.05
	***	***	***	***	***	NS
Sr. ALP	< 0.00001	< 0.00001	< 0.00001	>0.05	< 0.001	< 0.05
	***	***	***	NS	***	*
Cholesterol	< 0.00001	< 0.00001	< 0.00001	< 0.0001	< 0.001	>0.05
	***	***	***	***	***	NS
Triglyceride	< 0.00001	< 0.00001	< 0.00001	>0.05	>0.05	< 0.05
	***	***	***	NS	NS	*
Total protein	< 0.00001	< 0.00001	< 0.00001	>0.05	>0.05	>0.05
	***	***	***	NS	NS	NS
Albumin	< 0.00001	< 0.001	< 0.0001	>0.05	>0.05	>0.05
	***	***	***	NS	NS	NS
Globulin	< 0.00001	< 0.00001	< 0.0001	>0.05	>0.05	>0.05
	***	***	***	NS	NS	NS

TD1, Test drug 1; TD2, Test drug 2; NS, not significant; Sr. ALP, serum alkaline phosphatase; T. Protein, Total Protein.

^*^ Significant; ^**^ very significant; ^***^ highly significant.

#### Histopathology

**Figure 10:**
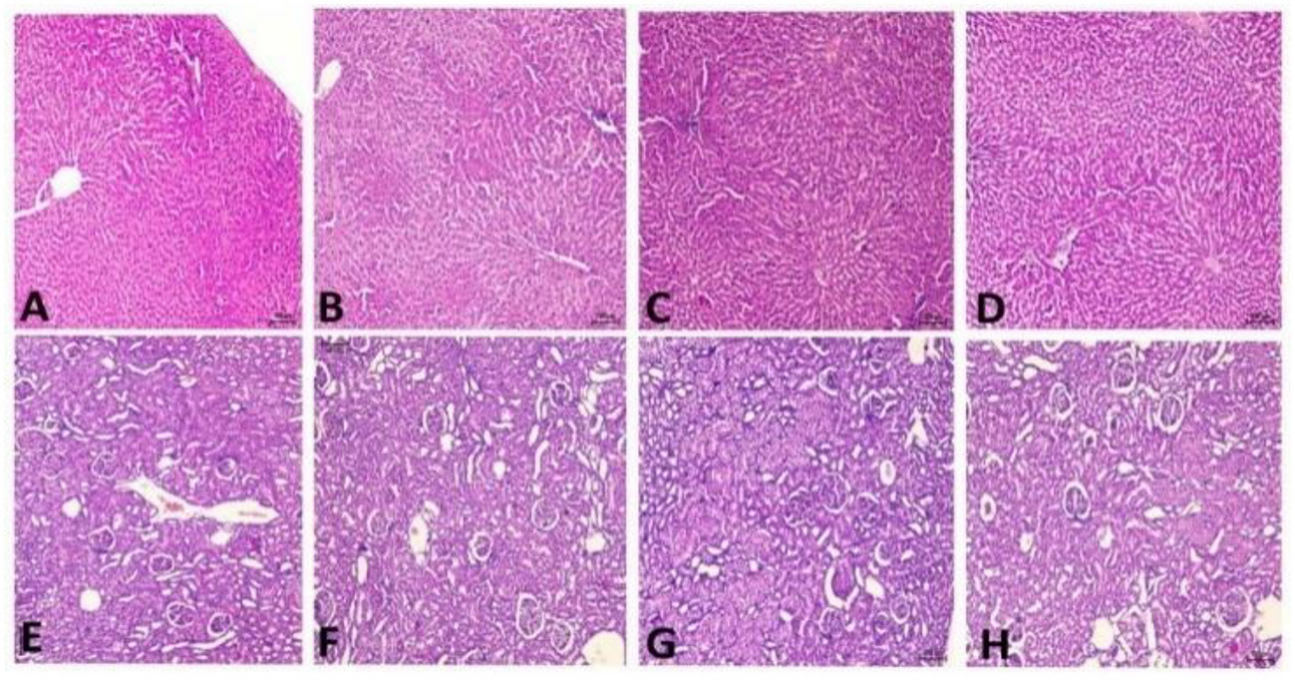
Histopathology reports. **(A)** Control group showing moderate lesions in the liver, **(B)** TD1 group showing mild lesions in the liver, **(C)** TD2 group showing minimal lesions in the liver, **(D)** Standard group showing minimal lesions in the liver, **(E)** Control group showing mild lesions in the kidney, **(F)** TD1 group showing mild to minimal lesions in the kidney, **(G)** TD2 groups showing minimal lesion in the kidney, **(H)** Standard group showed minimal lesions.

Histopathology changes in the liver and kidney of all groups are illustrated in Figure 10, and data of different groups are summarized in Table 10. Fatty accumulation, inflammatory cellular infiltration, degeneration changes, and necrosis of hepatic tissues caused due to alcohol intoxication were improved in the treatment group of both doses of *TC*. But higher dose, 400 mg/kg of *TC*, has shown tremendous potential in reversing hepatic lesions. There is no fatty cell accumulation, inflammatory cells, and only minimal degenerative changes comparable to that of Silymarin.

**Table 10:** Results of histopathology of liver.

**Group**	**Vascular tissue**	**Hepatocytes**	**Hepatic parenchyma**	**Inflammatory cellular changes**	**Overall pathological lesion score**
**Histopathological analysis—liver**					
Control	Focal congestion of central vein	Cellular swelling, granular cytoplasm, and presence of fatty accumulation. Mild degenerative changes and focal necrotic changes with loss of cellular border	Focal necrotic changes with loss of nucleus	Focal inflammatory cellular infiltration of mononuclear cells	Moderate (+3)
TD1	Focal congestion of central vein	Mild degenerative changes with granular cytoplasm and presence of fatty accumulation	Focal necrotic changes with loss of nucleus	Focal inflammatory cellular infiltration of mononuclear cells	Mild (+2)
TD2	No changes	Minimal degenerative changes with granular cytoplasmic changes with the occasional presence of fatty accumulation	Focal changes	Absent	Minimal (+1)
Standard	No changes	Minimal degenerative changes with granular cytoplasmic changes with the occasional presence of fatty accumulation	Focal changes	Absent	Minimal (+1)
**Histopathological analysis—kidney**						
Groups	Vascular tissue	Renal parnchyma	Tubuarepitelium	Renal tubules	Glomeruli	Overall Pathological lesion score
Control	Focal congestion in medulla and cortex	Focal interstitial hemorrhages	Cellular swelling and tubular degeneration with focal necrosis in the medullary region	Mild to moderate degenerative changes with granular cytoplasm and enlarged tubules	Focal hypertrophic features of glomeruli in the cortical region	Mild (+1)
TD1	Focal congestion in medulla and cortex	Focal interstitial hemorrhages	Focal area of cellular swelling and tubular degeneration with focal necrosis in the medullary region	Focal degenerative changes with granular cytoplasm and enlarged tubules	Focal hypertrophic features of glomeruli in the cortical region	Minimal (+1) to Mild (+2)
TD2	No changes	No changes	Focal areas of cellular swelling and tubular degeneration with focal necrosis and presence of an eosinophilic mass in the tubular lumen	No changes	No changes	Minimal (+1)
Standard	No changes	No changes	Focal and minimal changes with cellular swelling	No changes	No changes	Minimal (+1)

Overall Grade score as—NAD, no abnormality detected, minimal changes (+1), mild changes (+2), moderate changes (+3), severe changes (+4).

## Discussion

### Network pharmacology-based analysis

In the present study, we analyzed the candidate components and targets associated with *TC* with the aid of network pharmacology analysis and discovered the association between 1,127 PCs and their 2,765 protein targets. ADME screening has resulted in the identification of 418 active compounds referred to as PACs with the ability to regulate 1,005 protein targets. Metabolite inter-conversion enzymes are an enriched class of protein targets, where P35354 and P42574 emerged as major targets, being regulated by 13 PACs of *TC*. P34354 is a Prostaglandin G/H synthase 2 encoded by Prostaglandin-Endoperoxide Synthase 2 (PTGS2) and also known as cyclooxygenase-2 (COX-2). COX-2 is a crucial enzyme in the formation of prostaglandins and thus plays a significant role in the inflammatory responses. COX-2 has been shown to be involved in carcinogenesis *via* apoptosis inhibition, where selective inhibitors of COX-2 can provide chemoprevention in the case of liver tumors (56). In such a case, it may be assumed that these 13 regulators of PTGS2 could contribute to providing an anti-inflammatory effect of *TC via* reducing the production of prostaglandins, which promote inflammation. The other major protein target of *TC*, P42574 is caspase-3 that is involved in apoptosis execution. The association of caspases in liver disease and their inhibitors are well studied in relation to liver diseases (57). Thus, suggesting the high therapeutic relevance of *TC* in managing liver diseases. GO-based annotation of the protein targets revealed the synergistic role of *TC* in regulating a multitude of cellular functions to elicit its therapeutic effect.

The construction and analysis of ALD-specific networks highlight the therapeutic efficacy of the formulation in managing ALD *via* its 40 PCs pathway association of the protein targets of ALD-specific 40 PCs revealed that *TC* mainly regulates pathways associated with cancer and signaling processes like HIF-1, chemokine signaling, PI3K-Akt, etc. The role of these signaling pathways in the progression of ALD is well-known, for example, interactions among cytokines and chemokines play a significant role in disease development (58). Also, HIF-1α plays a crucial role in the development and prognosis of ALD, and targeting the same offers an attractive strategy for the treatment of ALD (59). This suggests that the enriched pathways might be the crucial pharmacological mechanism of *TC* for ALD management. Also, mapping of protein targets on the ALD-disease pathway reflects the direct regulation of *TC*. For example, Sirtuin 1 encoded by the SRT1 gene, is a target of *TC* whose reduced levels have been linked to the inflammatory processes observed in ALD patients. This study also highlighted the multi-targeting roles of PACs of *TC*, which offer an additional advantage to these PCs in future drug-discovery procedures for their multi-targeting ability toward ALD proteins. Phytochemical-specific regulatory potential of *TC* upon the ALD-specific Human-PIN was also identified, where both direct and indirect control of *TC* over the clusters were identified. It was found that direct control takes place through their protein targets and indirect *via* proteins interaction of the *TC* protein targets. The regulation over clusters was mainly through PI3K-Akt signaling, NF-kappa B signaling, and pathways involved in cancer, especially hepatocellular carcinoma. Inflammation plays a vital role in disease progression, leading to fibrosis, which can later develop into cirrhosis. As PI3K-Akt activates immune cells *via* regulating inflammatory cytokines, the regulation of this pathway may contribute to achieving therapeutic effects for ALD (55). Also, NF-kappa B performs a wide range of cellular functions, influencing inflammation in Kupffer cells and survival of hepatocytes, thus linking injury, fibrosis, and hepatocellular carcinoma (60). These data support the therapeutic efficacy of *TC* toward ALD.

### Physicochemical analysis

Foreign matter, moisture content, total ash value, acid insoluble ash, water-soluble ash, water-soluble extract, and alcohol soluble extract of *Z. officinale, P. longum*, and *P. nigrum* were found under the permissible limits of pharmacopeia standards. In crude drugs, a number of constituents are present, and they have the selective solubility rate in different solvents such as water and alcohol. Water can extract ether polar substances such as alkaloids, tannins, and flavonoids, but alcohol extracts the compounds that seem insoluble in water, such as sterols and phenols. Water and alcohol soluble extract content in *TC* is found to be more than that of their individual powder, indicating a higher concentration of substances in *TC*. As *TC* has higher concentration of substances, so it may show more activity than individual herbs. Combination of herbs are increasing its potency.

If maintained under the same conditions, HPTLC analysis shows that all the three batches of *TC* have an Rf value of 0.43, indicating the presence of *Piperine* (45–47). *Piperine* is an essential and major component of *TC* formulation and its presence in the sample suggests its standard quality. The fingerprinting of the *TC* presents that at Rf 0.80, all the three batches show a maximum peak, in the first batch maximum% age is demonstrated by 0.37 Rf i.e., to be 25.87%, in the second batch maximum% age is shown by 0.35 Rf i.e., to be 30.39%, in third batch maximum% age is shown by 0.35 Rf i.e., 30.20%. Second and third, both the batches show maximum% age of amount at Rf 0.35, and that is almost equal to 30%.

### Experimental analysis

#### Body weight

Liver diseases cause insufficient absorption and metabolism of nutrients resulting in malnutrition and decreased body weight. This study evidenced a significant increase in rats' weights among TD2, TD1, and standard groups which is not evident in the control group directed toward the reversal of liver injury by *TC* (61).

#### Biochemical parameters

Alcohol consumption causes substantial diseases, and the liver is the most adversely affected organ. Liver function test (LFT), is a well-known biochemical test that is used to determine the health and function of the liver through the measurement of liver enzymes (SGOT, SGPT, alkaline phosphate), bilirubin, and protein in the blood (62). During intoxication of alcohol, there are structural changes, leading to an increase in the permeability of the cell membrane. As there is an increase in permeability of the membrane, it further produces SGOT and SGPT leakage into blood circulation as shown by abnormally high levels of serum hepatic markers. Elevated LFT from the normal values is the indication of liver injury (62). *TC* shows a reduction in SGOT and SGPT levels in these respective studies of alcohol-mediated hepatotoxicity which may be attributed to its cell membrane stabilizing ability, hepatic enzymes leakage prevention, and translocation into the serum (63). Liver steatosis is the earliest stage of liver disease in chronic ethanol consumption, with the characteristic of fat accumulation (64). In this study, alcohol-induced hepatic steatosis was demonstrated by increased cholesterol, triglycerides values, and fatty accumulation in liver histology (64). Data from the present study the first show that *TC* can reverse the ethanol-induced hepatotoxicity. Oral treatment of *TC* by both doses significantly reduced the elevated biochemical markers compared with the control group. The highest suppressive effect on decreasing SGOT, SGPT, total bilirubin, and cholesterol was induced by the high dose of *TC* 400 mg, respectively, comparable to that of silymarin, and decreased triglyceride level more than that of silymarin.

The total protein content consists of globulin and albumin protein. Albumin is a very common protein found in the blood with various functions, produced by the liver only. The blood levels of these plasma proteins are decreased when there is extensive liver damage. Hypoalbuminaemia may occur in liver disease, with significant destruction of hepatocytes and hyperglobulinaemia in chronic inflammatory diseases such as cirrhosis and chronic hepatitis (64, 65). Usually, in liver diseases, synthesis of this protein decreases, but in this study after administration of ethanol, it is noted that the values of total protein, globulin, and albumin neither increased nor decreased, the values just are in the normal range. But after treatment, it was noticed that there is a difference in the means of each treatment. In all the three treatment groups-total protein, globulin, and albumin values are found significantly less compared to that of the control group also reflecting toward the improved functional capacity of the liver by *TC* (64).

#### Histopathology

Pretreatment with ethanol resulted in damage to the liver as infiltration of mononuclear cells, distortion of the normal structure of the hepatocytes cells, loss of nucleus, presence of necrotic cells, and accumulation of fatty cells were detected in many regions of the liver sections (66). Treatment with *TC* at both doses and silymarin has helped in modulating degenerative changes, hepatic lesions, inflammatory cellular infiltration, and fatty accumulation in hepatocytes which is evident in the control group. The higher dose of 400 mg/kg of *TC* showed a remarkable liver regeneration by improving hepatic lesions, inflammation, and the only occasional presence of fatty changes (66).

Histopathology of the kidney shows mild pathological changes in the control group, demonstrating harmful effects of chronic consumption of ethanol on the kidney. *TC*, however, also demonstrates improvement in pathological lesions of the kidney compared to that of silymarin indicating its renal protective ability (67).

## Possible scenario of hepatoprotection mechanism-*TC*

**Figure 11:**
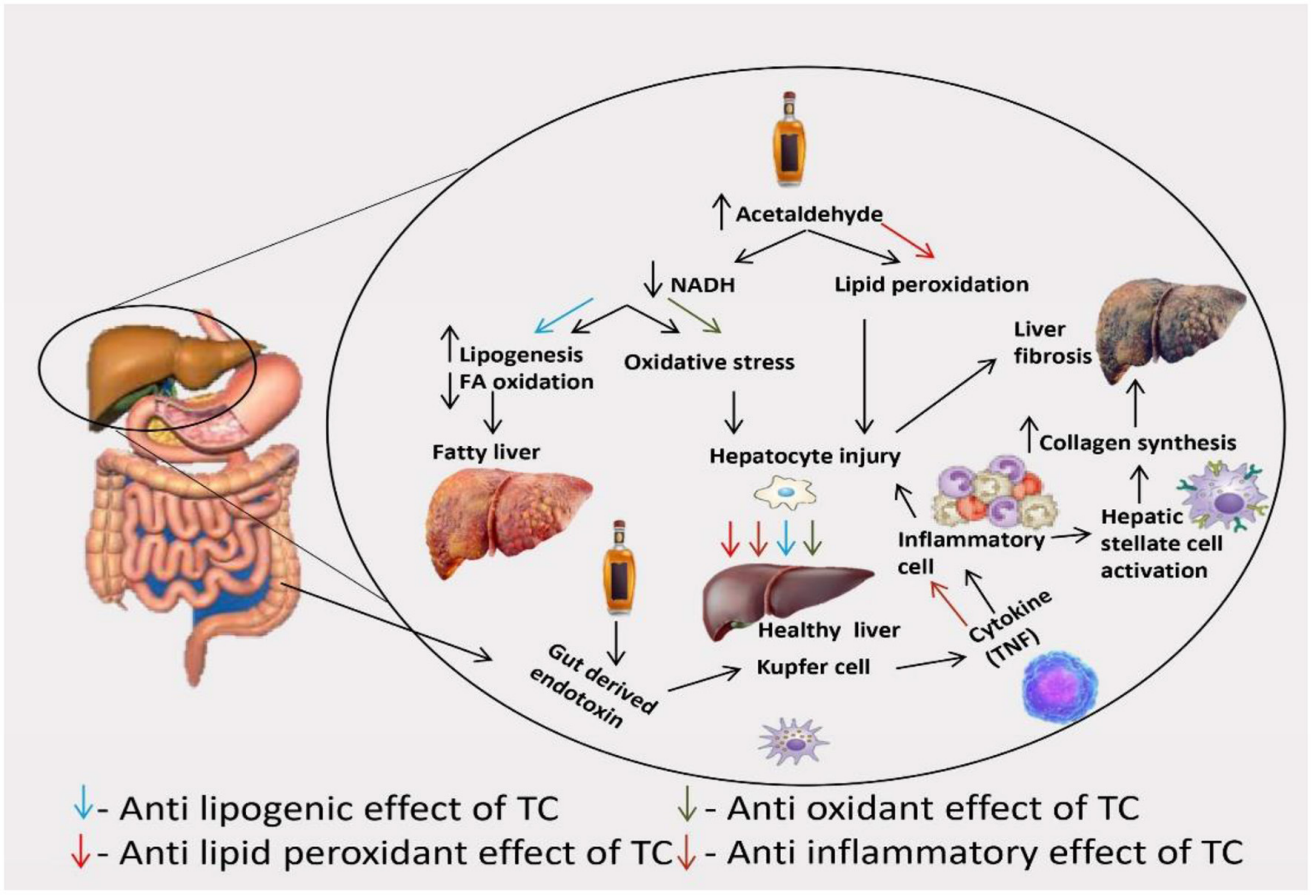
Mechanism of action of *TC* in management of alcohol-induced hepatotoxicity.

The exact pathogenesis of alcoholic liver injury is yet not known that why only some chronic alcoholics develop the complete sequence of changes in the liver while the others don't. Ethanol and its metabolites are responsible for ill effects on the liver. Biomedical and cellular events such as—changes in fat contents in the liver, cytoskeletal and membrane damage of a hepatic cell, hepatic fibrogenesis, oxidative damage to membrane and proteins, inflammatory reaction, activation of stellate cell and kupffer, lactic acidosis and collagen formation, which occurs due to chronic alcohol consumption culminate in morphologic lesions of alcoholic steatosis (fatty liver), alcoholic hepatitis and alcoholic cirrhosis, which is evidenced by elevated biochemical parameter SGOT, SGPT, ALP, total bilirubin, cholesterol, triglyceride level, and histopathology. *TC* helps in reducing the elevated biochemical parameters. Also, *TC* reduces oxidative stress, fatty changes, inflammatory reactions, and damage to hepatocytes; thus, acclimating to hepatoprotection. Previous studies also report their antioxidant and anti-inflammatory activity in numerous *in vivo* and *in vitro* models. The ability of *TC* to cope with the oxidative stress and inflammation induced by alcohol could be accountable to the antioxidant and anti-inflammatory capacity of the herb (14, 16, 20, 68). The association of protein targets of *TC* in metabolic pathways, signaling processes like HIF1α, PI3k-Akt, NF-kappa B, chemokine/cytokine, and apoptotic pathways as observed in network pharmacological analysis confirms its role in reducing inflammation, oxidative stress, and fibrosis. Especially the molecular targets of *TC* like PTGS2, SRT1, and caspases are of direct relevance in achieving its desired hepatoprotective effects. Most importantly, *TC* help to lower the level of liver enzymes in relieving the symptoms of ALD, and metabolic enzymes were recognized as the major target class of phytochemicals considering network analyses. The possible scenario of *TC* action in interfering with hepatoprotective activity is depicted in Figure 11.

## Limitations and future perspective

As many Rfs were found in fingerprinting, *TC* can be evaluated for these compounds by running with multiple standard markers in HPTLC or by other chromatographic tests. The process will also enable the quantification and standardized analysis of other compounds, allowing for a complete physicochemical profile to be generated. The quantification of each compound also plays a major role in its therapeutic effect. So, all the compounds present in the *TC* should be quantified in future studies to find out in which quantity these effects can be produced.

*TC*, its metabolites, or different extracts can be evaluated for its hepatoprotective effect by using different *in vivo* and *in vitro* hepatotoxicity models, multiple doses, chronic toxicity, and multi-centric clinical trials. Furthermore, the study indicates that *TC* and silymarin can improve kidney damage due to alcohol consumption. Therefore, both these drugs can be evaluated for the kidney protective effect in different experimental models and clinical trials, and therefore results can be validated. Also, through network pharmacology techniques, the possible involved mechanisms in the execution of this effect can also be ruled out.

The pathways detected in executing hepatoprotective effects should also be verified in more experimental studies. Pathway specific animal should be conducted to understand the clear mechanism and developing the novel treatment for ALD.

## Conclusion

Our research presents a novel and feasible approach for screening active ingredients of *TC* herb responsible for ALD treatment along with the mechanism involved. In this study, we adopted a systematic network pharmacology approach to investigate the hepatoprotective effects of *TC* in ALD and it indicates that the hepatoprotective effects of *TC* are manifested by the multitude of mechanisms involving regulating cancer and signaling processes like AGE-RAGE signaling, HIF-1 signaling, NF-Kappa B signaling, PI3K/Akt signaling responsible for anti-inflammatory, anti-oxidant and anti-apoptotic role. Furthermore, *in vivo* experiments validate the therapeutic effects of *TC* at doses of 200 and 400 mg/kg. *TC* improved ALD symptoms by inhibiting inflammation, inhibiting cellular evasion, reducing fatty cell accumulation, and lowering liver enzymes. *TC* high dose of 400 mg/kg showed comparable effects to silymarin, but less at a dose of 200 mg/kg. In addition, *TC* also showed renal protective action by ameliorating kidney lesions caused due to ethanol.

## Data availability statement

The original contributions presented in the study are included in the article/Supplementary material, further inquiries can be directed to the corresponding authors.

## Ethics statement

An animal study was conducted at Agharkar Research Institute, Pune, India, according to the recommendations and approval of the Institutional Animal Ethical Committee with approval number ARI/IAEC/2019/11.

## Author contributions

All authors listed have made a substantial, direct, and intellectual contribution to the work and approved it for publication.

## Funding

This work was supported by the National Natural Science Foundation of China (Grant Nos. 32070671 and 32270690) and Regional Innovation Cooperation between Sichuan and Guangxi provinces (Project No. 2020YFQ0019).

## Conflict of interest

The authors declare that the research was conducted in the absence of any commercial or financial relationships that could be construed as a potential conflict of interest.

## Publisher's note

All claims expressed in this article are solely those of the authors and do not necessarily represent those of their affiliated organizations, or those of the publisher, the editors and the reviewers. Any product that may be evaluated in this article, or claim that may be made by its manufacturer, is not guaranteed or endorsed by the publisher.

## Supplementary material

Supplementary Figure S1 Trikatu-protein target (T-PT) Network: Network of size 2,963 nodes 7,055 edges, containing the information of 198 phytochemicals (cyan colored diamond nodes) and their 2,765 protein targets (pink colored circular nodes). The size of the nodes varies according to their degree value in the net-work, where node with the highest degree is bigger in size corresponding to the other nodes. The protein targets were compiled from STITCH having interaction score ≥700.

Supplementary Figure S2 Liver specific disease gene (LD-G) Network. The proteins and their associated liver Disease term was used for obtaining liver specific disease gene (LD-G) Network, as per the curated gene-disease pair data available in DisGeNET. LD-G network comprise of 539 nodes (515 proteins and 24 disease terms) and 1,308 edges, where pink nodes are specific to proteins and green denote disease class.

Supplementary Table S1 38 liver disease terms considered in the study (as per DisGeNET data).

Supplementary Table S2 Trikatu-protein target (T-PT) network specific to PACs.

Supplementary Table S3 Protein class association using PANTHER.

Supplementary Table S4 Disgnet disease-protein association data corresponding to Liver diseases (LD-G network).

Supplementary Table S5 Protein targeting ability of PCs in the ALD-PT network.

Supplementary Table S6 KEGG pathway enrichment.

Supplementary Table S7 Network analysis of TRH-PIN.

Supplementary Table S8 MCODE cluster information along with the GO-based annotation and protein-class enrichment.
