# Supplementary figures and images for: Deciphering the impact and mechanism of Trikatu, a spices-based formulation on alcoholic liver disease employing network pharmacology analysis and in vivo validation
Source: Front Nutr. 2022 Nov 16;9:1063118. doi: 10.3389/fnut.2022.1063118 (PMC9709420; doi:10.3389/fnut.2022.1063118)

## Slide 1
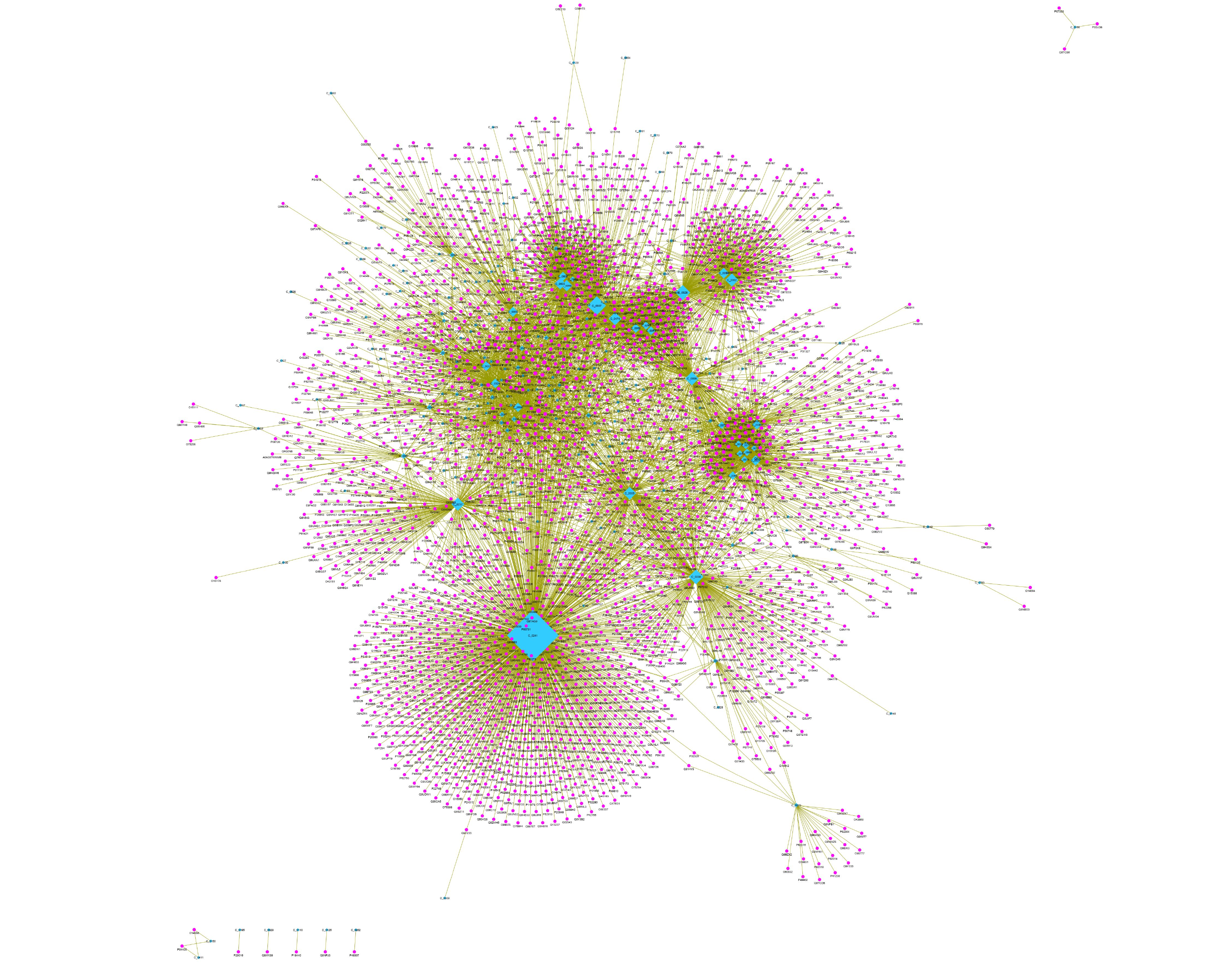

Supplement: Supplementary Figure S1 — Trikatu-protein target (T-PT) Network: Network of size 2,963 nodes 7,055 edges, containing the information of 198 phytochemicals (cyan colored diamond nodes) and their 2,765 protein targets (pink colored circular nodes). The size of the nodes varies according to their degree value in the net-work, where node with the highest degree is bigger in size corresponding to the other nodes. The protein targets were compiled from STITCH having interaction score ≥700. [file Data_Sheet_1.ZIP › Supplementary Figure 1.pptx]

## Slide 1
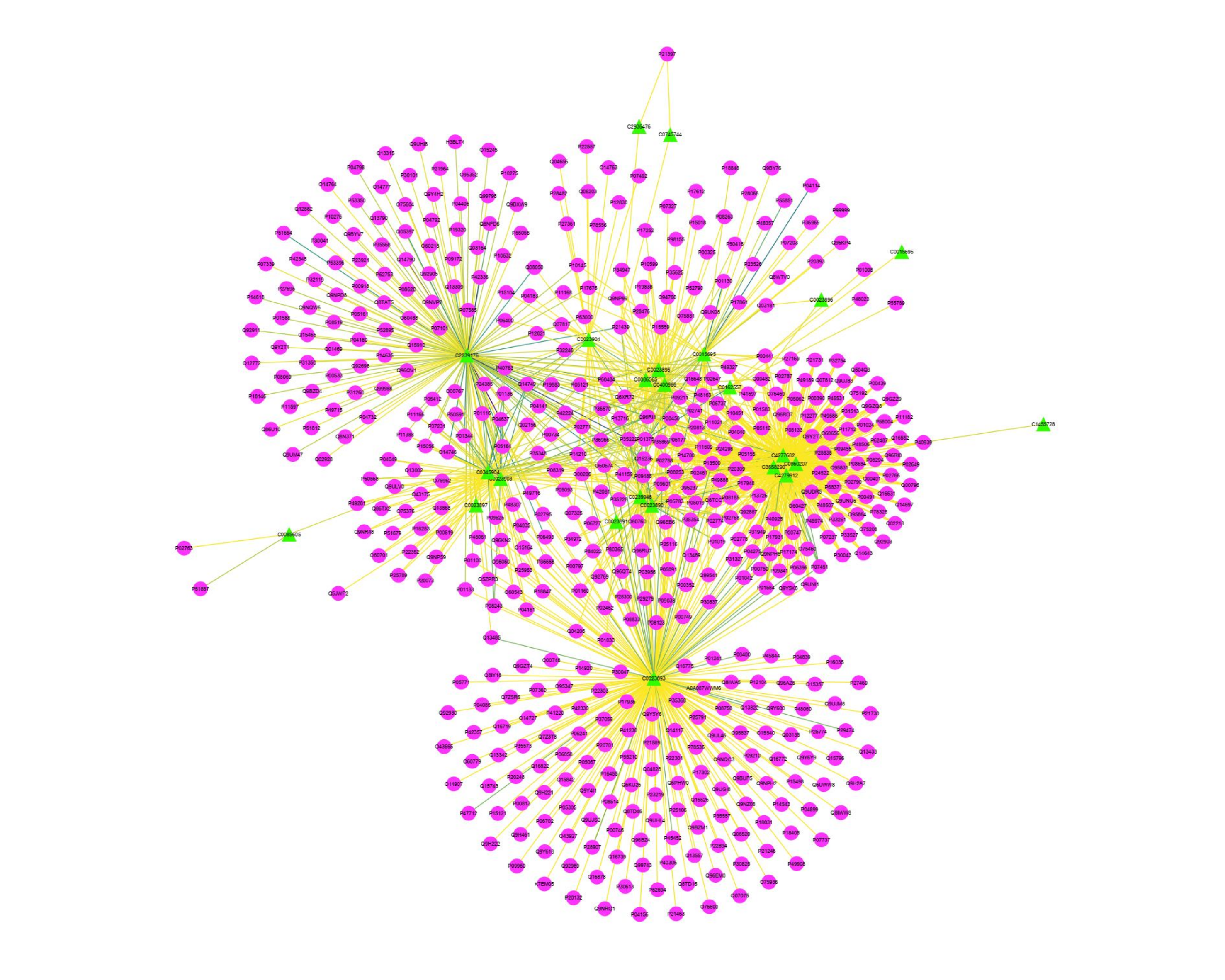

Supplement: Supplementary Figure S1 — Trikatu-protein target (T-PT) Network: Network of size 2,963 nodes 7,055 edges, containing the information of 198 phytochemicals (cyan colored diamond nodes) and their 2,765 protein targets (pink colored circular nodes). The size of the nodes varies according to their degree value in the net-work, where node with the highest degree is bigger in size corresponding to the other nodes. The protein targets were compiled from STITCH having interaction score ≥700. [file Data_Sheet_1.ZIP › Supplementary Figure 2 .pptx]
